# Supplementary material for: Single-cell multi-omics data reveal heterogeneity in liver tissue microenvironment induced by hypertension
Source: Mol Ther Nucleic Acids. 2025 Aug 23;36(4):102696. doi: 10.1016/j.omtn.2025.102696 (PMC12450633; doi:10.1016/j.omtn.2025.102696)
Supplement: Document S1. Figures S1–S5 and Tables S1, S4, S6, S8, and S12–S14 [file mmc1.pdf]

OMTN, Volume *36*

Supplemental information

Single-cell multi-omics data reveal heterogeneity in liver tissue microenvironment induced by hypertension

Hongfei Li, Lingyu Cui, Murong Zhou, Quan Zou, Yuming Zhao, Hao Lin, Yingjian Liang, Alfred Wei Chieh Kow, and Guohua Wang

Supplemental Figures

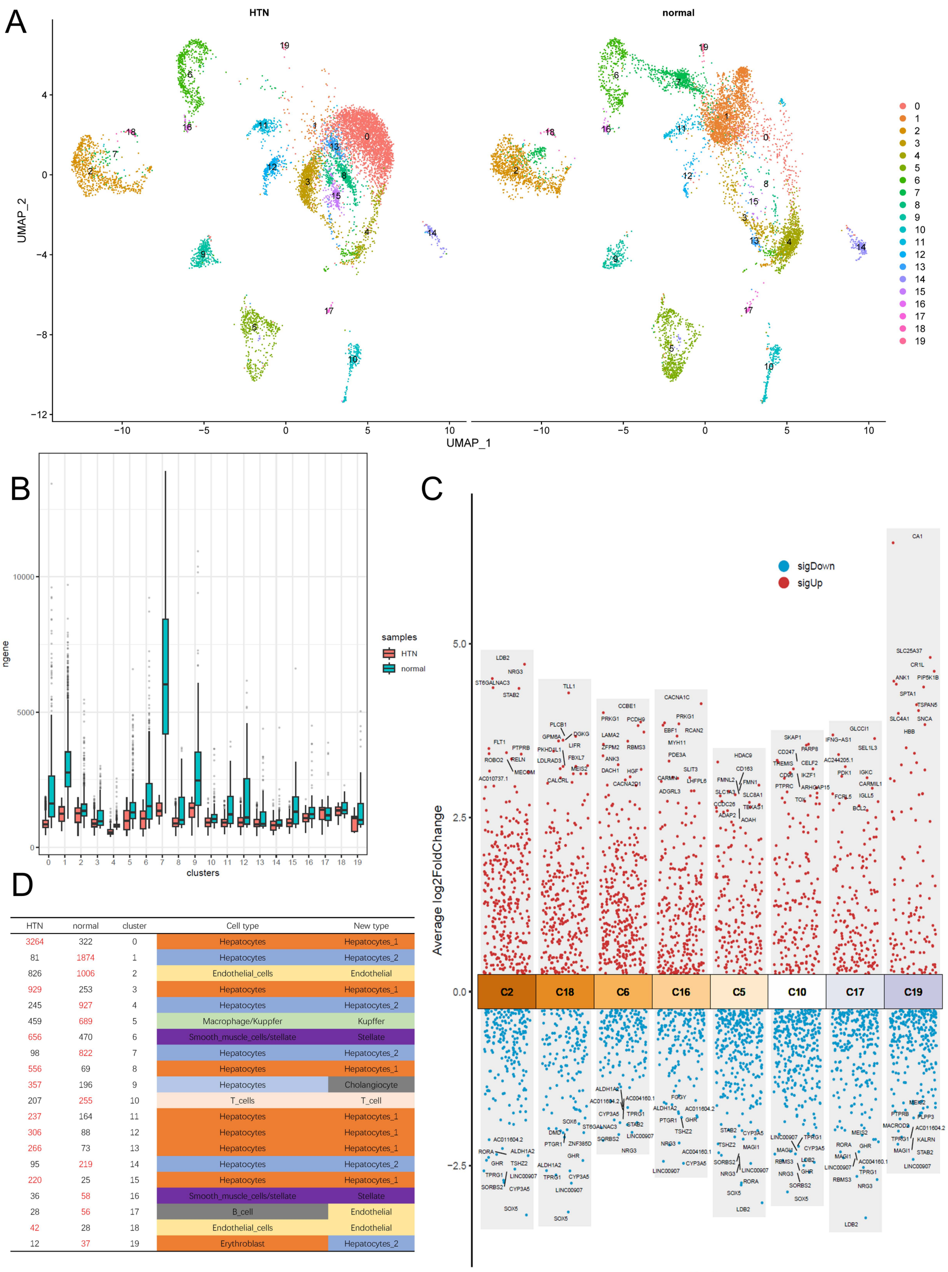

HTN	normal	cluster	Cell type	New type
3264	322	0	Hepatocytes	Hepatocytes_1
81	1874	1	Hepatocytes	Hepatocytes_2
826	1006	2	Endothelial_cells	Endothelial
929	253	3	Hepatocytes	Hepatocytes_1
245	927	4	Hepatocytes	Hepatocytes_2
459	689	5	Macrophage/Kuppfer	Kupffer
656	470	6	Smooth_muscle_cells/stellate	Stellate
98	822	7	Hepatocytes	Hepatocytes_2
556	69	8	Hepatocytes	Hepatocytes_1
357	196	9	Hepatocytes	Cholangiocyte
207	255	10	T_cells	T_cell
237	164	11	Hepatocytes	Hepatocytes_1
306	88	12	Hepatocytes	Hepatocytes_1
266	73	13	Hepatocytes	Hepatocytes_1
95	219	14	Hepatocytes	Hepatocytes_2
220	25	15	Hepatocytes	Hepatocytes_1
36	58	16	Smooth_muscle_cells/stellate	Stellate
28	56	17	B_cell	Endothelial
42	28	18	Endothelial_cells	Endothelial
12	37	19	Erythroblast	Hepatocytes_2

Figure S1. Heterogeneity of scRNA clusters

(A) UAMP of cell clustering for HTN and normal cells.

(B) Variation in the number of genes.

(C) Differential genes in hepatocyte subsets were excluded.

(D) Cell annotation based on singeR and manual.

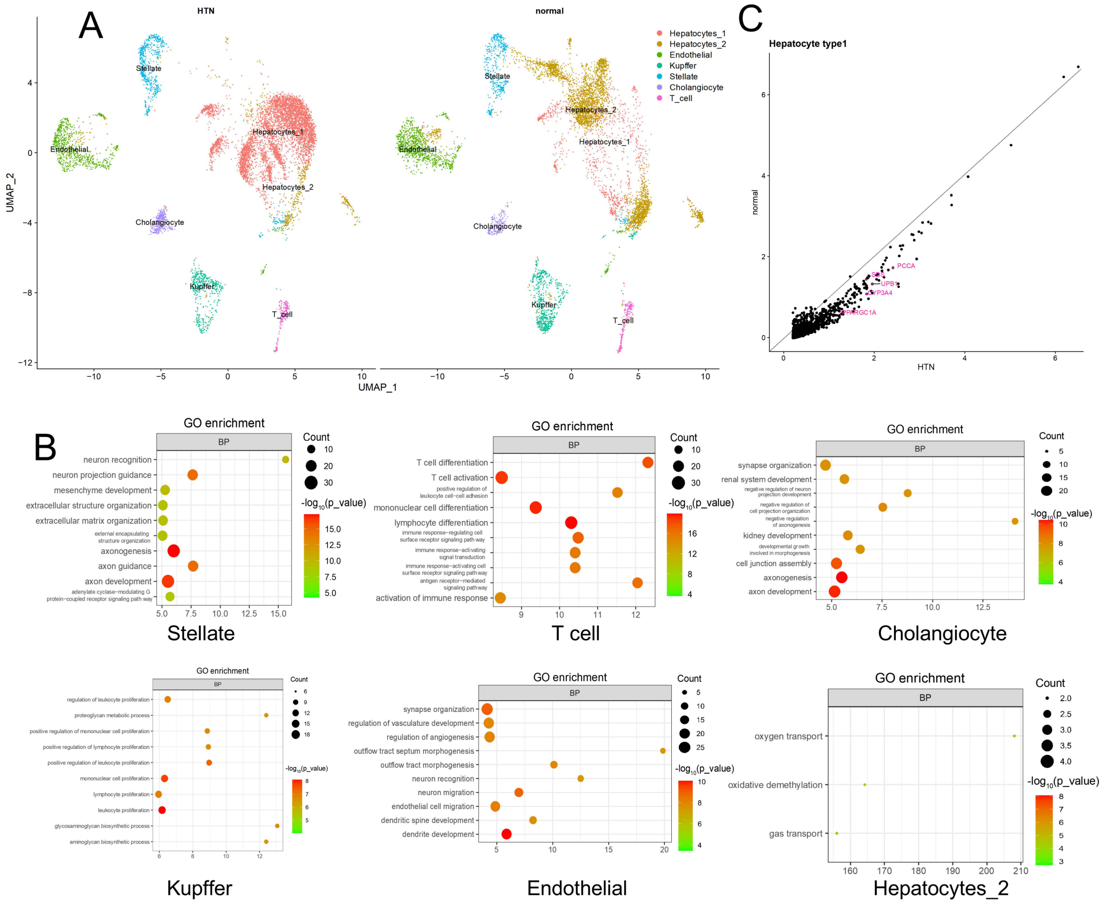

Figure S2. Functional enrichment of cell subsets

(A) UAMP of cell subsets for HTN and normal cells based on manual annotation.

(B) Enrichment of GO function in cell subsets.

(C) Differential expression of marker genes in hepatocytes_1.

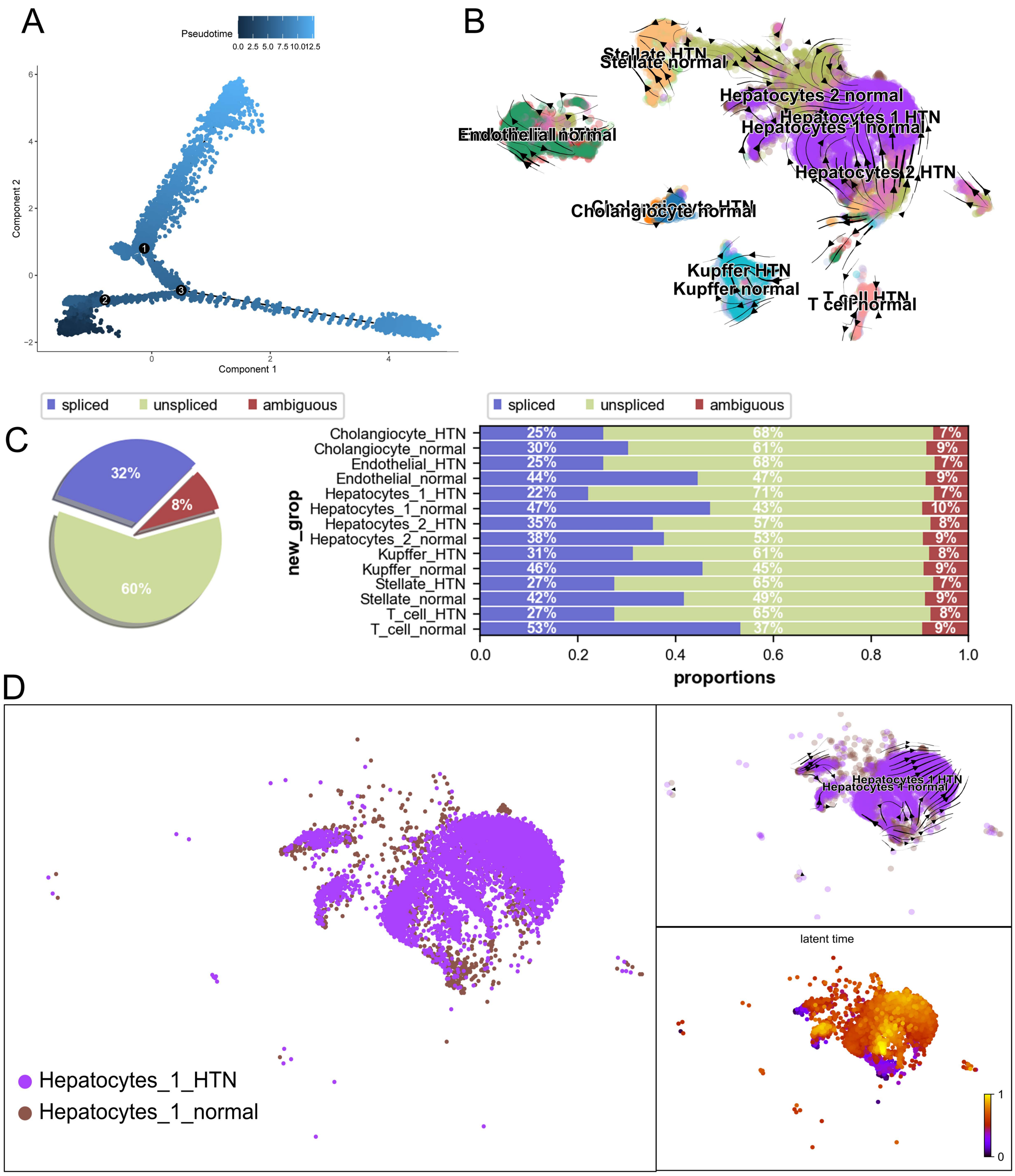

Figure S3. Validation of trajectory inference was performed using scVelo

(A) The pseudotime for trajectory inference in Monocle2.

(B). The RNA velocity trends of all cell types.

(C). The splicing states of all cell types.

(D). RNA Velocity Reveals the Directionality of Hepatocyte Differentiation Trajectory.

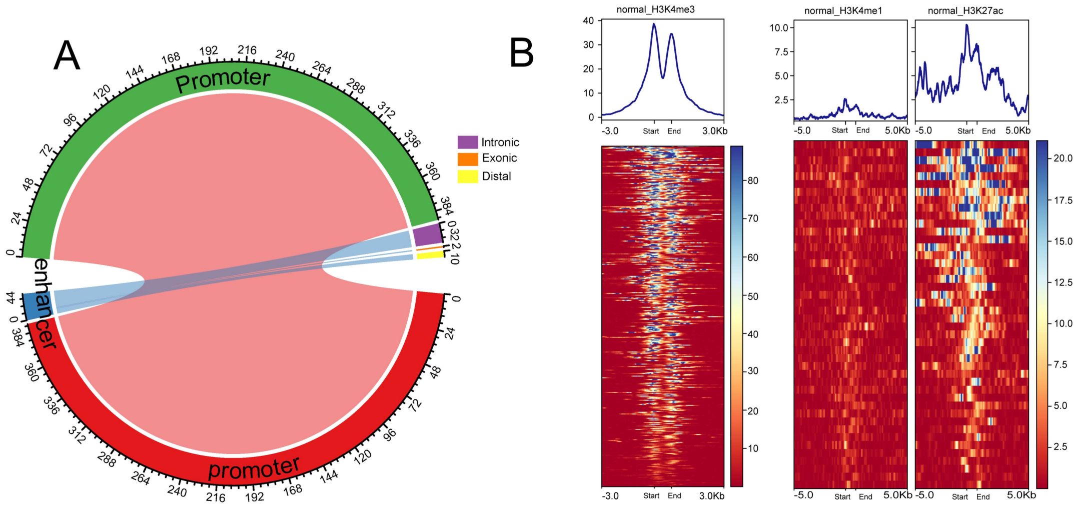

Figure S4. Predicting promoters of enhancers and promoters for normal Hepatocytes_1

(A) Annotation of promoter and enhancer for normal Hepatocytes_1.

(B). The distribution of histone signals in the promoter and enhancer regions of normal Hepatocytes_1.

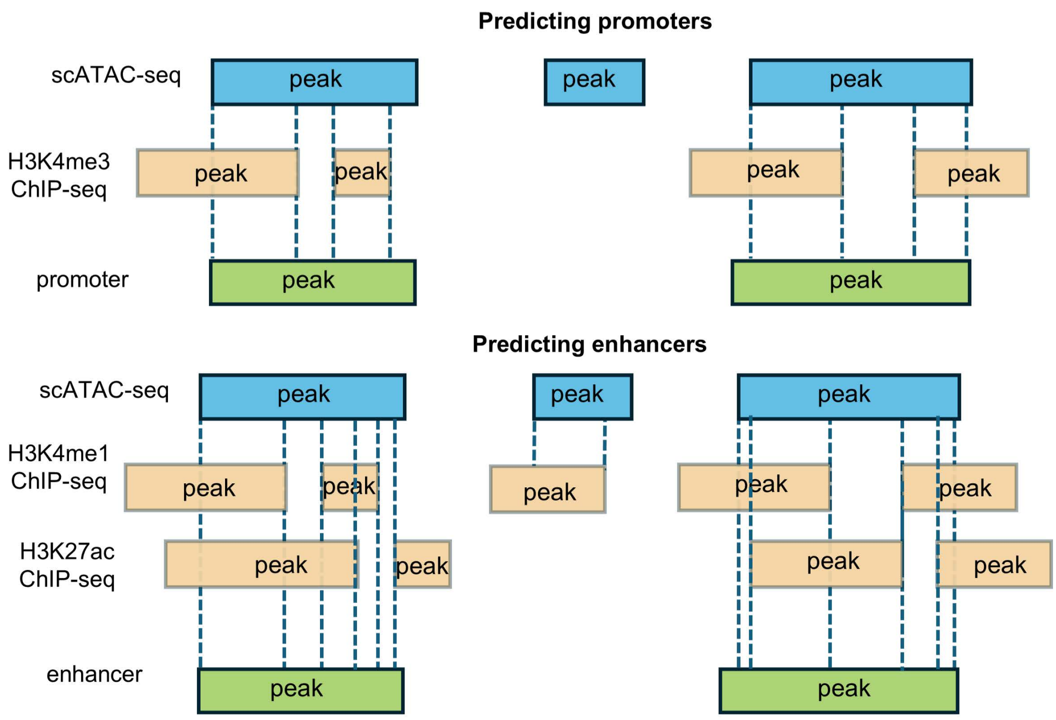

Figure S5. Predicting promoters and enhancers by overlapping peaks from scATAC-seq and histone ChIP-seq data.

Supplemental Tables

Table S1. The result of clusters and their corresponding cell numbers from the initial clustering of scRNA-seq.

HTN	normal	cluster	Percentage
3264	322	0	0.216663646
81	1874	1	0.118119751
826	1006	2	0.110688176
929	253	3	0.071415624
245	927	4	0.070811431
459	689	5	0.069361368
656	470	6	0.068032143
98	822	7	0.055585765
556	69	8	0.037762069
357	196	9	0.033411878
207	255	10	0.027913721
237	164	11	0.024228143
306	88	12	0.023805208
266	73	13	0.020482146
95	219	14	0.018971663
220	25	15	0.014802731
36	58	16	0.005679415
28	56	17	0.005075222
42	28	18	0.004229352
12	37	19	0.002960546

Table S2. Differential genes from the initial clustering of scRNA-seq (see Excel file).

Table S3. Average expression values of 2,000 genes in the finally annotated cell types (see Excel file).

Table S4. The number of clusters and corresponding cell counts from the annotated cell types of scRNA-seq.

HTN	normal	cluster
5778	994	Hepatocytes_1
531	3879	Hepatocytes_2
896	1090	Endothelial
459	689	Kupffer
692	528	Stellate
357	196	Cholangiocyte
207	255	T_cell

Table S5. Differential genes from the finally annotated cell types in scRNA-seq (see Excel file).

Table S6. The enriched GO pathways based on upregulated genes (avg_log2FC > 0 and p_val < 0.05) of Hepatocytes_1 base on Table S5.

ONTOLOGY	ID	Description	GeneRatio	BgRatio	pvalue	p.adjust	qvalue	geneID	Count
BP	GO:0006631	fatty acid metabolic process	10/67	390/18723	0.00000118	0.00198	0.001573337	ECHDC2/PTGR1/PCCA/CYP3A4/ PLIN5/PNPLA3/CYP2C19/PPARGC1A/PCK1/MLXIPL	10
BP	GO:0044282	small molecule catabolic process	9/67	376/18723	0.00000739	0.00413	0.00328205	ECHDC2/PCCA/KMO/CYP3A4 /UPB1/PLIN5/GOT1/SDS/PCK1	9
BP	GO:0042445	hormone metabolic process	7/67	218/18723	0.0000127	0.004625	0.003675628	ALDH1A2/GHR/CYP3A5/PCSK6/ SLC16A10/CYP3A4/PPARGC1A	7
BP	GO:0046394	carboxylic acid biosynthetic process	8/67	314/18723	0.0000158	0.004625	0.003675628	ALDH1A2/KMO/NR1H4/CYP3A4/ UPB1/GOT1/SDS/MLXIPL	8
BP	GO:0016053	organic acid biosynthetic process	8/67	316/18723	0.0000165	0.004625	0.003675628	ALDH1A2/KMO/NR1H4/CYP3A4/ UPB1/GOT1/SDS/MLXIPL	8
BP	GO:0046395	carboxylic acid catabolic process	7/67	236/18723	0.0000211	0.004695	0.003730857	ECHDC2/PCCA/KMO/ PLIN5/GOT1/SDS/PCK1	7
BP	GO:0016054	organic acid catabolic process	7/67	240/18723	0.0000236	0.004695	0.003730857	ECHDC2/PCCA/KMO/ PLIN5/GOT1/SDS/PCK1	7
BP	GO:0006721	terpenoid metabolic process	5/67	97/18723	0.0000252	0.004695	0.003730857	ALDH1A2/CYP3A5/ EGFR/CYP3A4/CYP2C19	5
BP	GO:0006090	pyruvate metabolic process	5/67	106/18723	0.0000387	0.005693	0.004523819	SLC4A4/SDS/ PPARGC1A/PCK1/MLXIPL	5
BP	GO:0071466	cellular response to xenobiotic stimulus	6/67	177/18723	0.0000406	0.005693	0.004523819	RORA/CYP3A5/EGFR/ UGT1A10/CYP3A4/CYP2C19	6
BP	GO:0006805	xenobiotic metabolic process	5/67	111/18723	0.0000482	0.00622	0.004942357	RORA/CYP3A5/UGT1A10/ CYP3A4/CYP2C19	5
BP	GO:0006720	isoprenoid metabolic process	5/67	116/18723	0.0000595	0.007129	0.00566472	ALDH1A2/CYP3A5/EGFR/ CYP3A4/CYP2C19	5
BP	GO:0071375	cellular response to peptide hormone stimulus	7/67	290/18723	0.0000782	0.00874	0.006945063	GHR/BRIP1/ERRFI1/GOT1/ PNPLA3/TSHR/PCK1	7

Table S7. Differential genes between HTN and normal states of Hepatocytes_1 (see Excel file).

Table S8. The numbers of the four types of peaks in Hepatocytes_1 under HTN and normal conditions.

Peak type	normal	HTN
Distal	2547	1586
Exonic	500	717
Intronic	2993	3147
Promoter	547	1102
Total	6587	6552

Table S9. Predicted promoters of Hepatocytes_1 in the HTN state (see Excel file).

Table S10. Predicted enhancers of Hepatocytes_1 in the HTN state (see Excel file).

Table S11. Predicted promoters of Hepatocytes_1 in the normal state (see Excel file).

Table S12. Predicted enhancers of Hepatocytes_1 in the normal state.

Chromosome	Start	End	Peak ID	Gene	ArchR-annotated peak types
chr1	39927586	39928086	4215	MYCL	Distal
chr1	40383720	40384220	4274	SMAP2	Intronic
chr1	201512891	201513391	12449	CSRP1	Distal
chr2	73032654	73033154	20384	SFXN5	Intronic
chr2	177239905	177240405	25213	MIR3128	Intronic
chr2	224379425	224379925	27479	FAM124B	Exonic
chr3	46868180	46868680	31630	PTH1R	Intronic
chr3	113686109	113686609	34639	USF3	Intronic
chr4	6918130	6918630	40200	TBC1D14	Intronic
chr4	184345571	184346071	46757	LINC0236	Intronic
chr5	67547321	67547821	49779	LOC10192	Distal
chr5	150515886	150516386	53991	NDST1-AS	Intronic
chr5	150589369	150589869	54011	SYNPO	Distal
chr5	151085368	151085868	54084	TNIP1	Intronic
chr5	176534709	176535209	55389	RNF44	Intronic
chr6	53301237	53301737	60078	MIR5685	Intronic
chr6	169905609	169906109	65247	LINC0024	Distal
chr9	38389178	38389678	83112	ALDH1B1	Distal
chr9	104911079	104911579	85349	ABCA1	Intronic
chr9	129570304	129570804	87510	NTMT1	Distal
chr10	102280120	102280620	95426	GBF1	Intronic
chr11	86940924	86941424	103312	FZD4	Intronic
chr12	29241644	29242144	107514	LOC10050	Intronic
chr12	50304106	50304606	108343	LIMA1	Distal
chr12	111777634	111778134	111708	MIR6761	Intronic
chr13	97257670	97258170	115927	MBNL2	Intronic
chr13	110507025	110507525	116556	COL4A2-A	Intronic
chr15	63211400	63211900	124029	RAB8B	Intronic
chr15	78880941	78881441	125381	CTSH	Intronic
chr15	90035450	90035950	126062	MIR3174	Intronic
chr16	79970435	79970935	131723	MAFTRR	Distal
chr16	81552895	81553395	131848	MIR7854	Intronic
chr17	29153763	29154263	135200	MYO18A	Intronic
chr17	40108171	40108671	136063	NR1D1	Distal
chr17	64085049	64085549	138108	ERN1	Intronic
chr17	64156000	64156500	138128	SNORA50	Intronic
chr18	44725573	44726073	142172	SETBP1	Intronic
chr19	7258252	7258752	145241	INSR	Intronic
chr19	15325110	15325610	146300	BRD4	Intronic
chr19	18496409	18496909	146862	ELL	Intronic
chr19	38837709	38838209	147918	ECH1	Intronic
chr19	46975156	46975656	148960	NPAS1	Intronic
chr22	37503824	37504324	158423	CARD10	Exonic
chr22	46058088	46058588	159505	PRR34	Intronic

Table S13. Regulatory network of promoters of upregulated genes in HTN-Hepatocytes_1.

Promoter (peak ID)	Gene	TF
58694	ANKS1A	EBF1
58694	ANKS1A	WT1
58694	ANKS1A	ETS1
58694	ANKS1A	EHF
58694	ANKS1A	FLI1
58694	ANKS1A	ELF3
58694	ANKS1A	PAX2
70606	CYP3A4	FOXP1
70606	CYP3A4	GATA4
70606	CYP3A4	PBX1
70606	CYP3A4	RORA
70606	CYP3A4	RORB
85666	PALM2-AKAP2	WT1
131100	PDXDC2P-NPIPB14P	EBF1
131100	PDXDC2P-NPIPB14P	WT1
131100	PDXDC2P-NPIPB14P	ETS1
131100	PDXDC2P-NPIPB14P	EHF
131100	PDXDC2P-NPIPB14P	FLI1
131100	PDXDC2P-NPIPB14P	ELF3
131210	TAT-AS1	EBF1
131210	TAT-AS1	MEF2C
131210	TAT-AS1	RARB
131210	TAT-AS1	TCF7L1
136475	G6PC	HNF1B
136475	G6PC	RORA
136475	G6PC	TCF7L1

Table S14. Regulatory network of enhancers of upregulated genes in HTN-Hepatocytes_1.

Region	Gene	TF	Peak ID	data_attributes
chr13:94157698-94158198	GPC6	CEBPD	115803	genehancer_id=GH13F094157
chr22:37178198-37178698	IL2RB	E2F8	158352	genehancer_id=GH22F037175
chr22:24510675-24511175	UPB1	NR1H4	157194	genehancer_id=GH22F024510
chr22:24510675-24511175	UPB1	THRB	157194	genehancer_id=GH22F024510
chr5:150142139-150142639	CSF1R	NR1H4	53922	genehancer_id=GH05F150133
chr5:150142139-150142639	PDGFRB	THRB	53922	genehancer_id=GH05F150133
chr3:43317938-43318438	SNRK	NR2F1	31315	genehancer_id=GH03F043316
chr3:43317938-43318438	SNRK	NR2F2	31315	genehancer_id=GH03F043316
chr3:43317938-43318438	SNRK	THRB	31315	genehancer_id=GH03F043316
chr9:35834520-35835020	TPM2	NR2F1	82894	genehancer_id=GH09F035831
chr5:157288526-157289026	ITK	NR2F2	54386	genehancer_id=GH05F157287
chr5:157288526-157289026	ITK	PPARG	54386	genehancer_id=GH05F157287
chr10:112261401-112261901	GPAM	PPARA	96006	genehancer_id=GH10F112259
chr8:124630983-124631483	MTSS1	ZNF385D	79770	genehancer_id=GH08F124625
chr10:88840070-88840570	LIPN	ELK3	94265	genehancer_id=GH10F088840
chr6:39280222-39280722	KCNK17	PRRX1	59150	genehancer_id=GH06F039279
chr12:113246398-113246898	TPCN1	NR2E3	111827	genehancer_id=GH12F113235
chr2:102120584-102121084	IL1R1	ETS1	21784	genehancer_id=GH02F102120
