# Supplementary material for: Single-cell multi-omics data reveal heterogeneity in liver tissue microenvironment induced by hypertension
Source: Mol Ther Nucleic Acids. 2025 Aug 23;36(4):102696. doi: 10.1016/j.omtn.2025.102696 (PMC12450633; doi:10.1016/j.omtn.2025.102696)
Supplement: Document S2. Article plus supplemental information [file mmc9.pdf]

Molecular Therapy
Nucleic Acids
Original Article

Single-cell multi-omics data reveal heterogeneity in liver tissue microenvironment induced by hypertension

Hongfei Li,[1,3] Lingyu Cui,[2] Murong Zhou,[2] Quan Zou,[1,3] Yuming Zhao,[2] Hao Lin,[4] Yingjian Liang,[5] Alfred Wei Chieh Kow,[6,7,8,9] and Guohua Wang[2]

[1]Yangtze Delta Region Institute (Quzhou), University of Electronic Science and Technology of China, Quzhou 324003, China; [2]College of Computer and Control Engineering, Northeast Forestry University, Harbin 150040, China; [3]Institute of Fundamental and Frontier Sciences, University of Electronic Science and Technology of China, Chengdu 610054, China; [4]School of Life Science and Technology, Center for Informational Biology, University of Electronic Science and Technology of China, Chengdu 610054, China; [5]Key Laboratory of Hepatosplenic Surgery, Ministry of Education, Department of General Surgery, the First Affiliated Hospital of Harbin Medical University, Harbin 150007, China; [6]Division of Hepatobiliary & Pancreatic Surgery, Department of Surgery, National University Hospital Singapore, Singapore 119074, Singapore; [7]Department of Surgery, Yong Loo Lin School of Medicine, National University of Singapore, Singapore 119077, Singapore; [8]Division of Surgical Oncology, National University Cancer Institute Singapore (NCIS), National University Health System, Singapore 119228, Singapore; [9]National University Digestive Center (NUCD), National University Health System Singapore, Singapore 119074, Singapore

Hypertension (HTN) may induce liver damage; however, the effects on liver cell subpopulations remain obscure. Understanding these microenvironmental changes could offer early HTN and liver disease indicators. We employed single-cell multi-omics and histone chromatin immunoprecipitation sequencing (ChIP-seq) to scrutinize microenvironmental alterations between normal and HTN liver tissues. Our analysis revealed an HTN-related hepatocyte subpopulation, termed "Hepatocytes_1," via single-cell RNA sequencing (scRNA-seq). Five potential pathogenic genes (UPB1, SDS, PCCA, CYP3A4, and PPARGC1A) were identified in Hepatocytes_1. Additionally, the regulatory network of *cis*-regulatory elements (CREs) in genes within Hepatocytes_1 was unveiled using single-cell assay for transposase accessible chromatin (ATAC) sequencing (scATAC-seq) and histone ChIP-seq. Specifically, the active promoter of the disease-associated gene CYP3A4 formed a transcription factor (TF)-mediated regulatory network, resulting in heightened expression in HTN cells. The enhancer-mediated regulatory relationship between NR1H4 and UPB1 serves as a potential regulator in maintaining hepatic metabolic homeostasis in response to HTN-induced disturbances. The identification of HTN-related gene markers and CREs in the liver provides novel insights for HTN prevention and therapeutic targeting.

INTRODUCTION

Hypertension (HTN) is a chronic disease with a mortality rate comparable to infectious diseases.[1] There are approximately one billion patients with HTN worldwide, accounting for one-eighth of the global population.[2] Cardiovascular diseases related to HTN are a leading cause of mortality among patients.[3] Given that the liver receives 25% of its blood supply from the heart, it is highly susceptible to fluctuations in blood pressure and blood flow, which explains why cardiovascular diseases can damage the liver.[4] Despite the manageability of HTN with medication, its impact on the cardiovascular system and liver tissue should not be underestimated.[2,3,5] Research indicates that approximately 50% of patients with HTN also have nonalcoholic fatty liver disease, although the exact causal relationship between HTN and liver disease remains unclear.[6–8] Therefore, understanding alterations in the liver tissue microenvironment caused by HTN is important for the prevention and treatment of both HTN and liver diseases.

Single-cell multi-omics technology can capture the distribution of omics signals within cell subpopulations, revealing disease-specific cell clusters and biomarkers.[9] Consequently, these technologies provide immense value for understanding disease pathogenesis and prevention.[10–13] Single-cell RNA sequencing (scRNA-seq) is instrumental in identifying disease-causing genes and is widely used to analyze the heterogeneity of the tumor microenvironment, providing valuable insights for targeted cancer therapies.[14–17] In the liver, scRNA-seq meticulously maps cellular composition,[18–23] revealing liver-specific sinusoidal endothelial cell types associated with portal HTN.[24] While scRNA-seq holds promise for discovering liver cell subpopulations and marker genes associated with HTN, the regulatory mechanisms of these genes are intricate and multidimensional. Single-omics approaches have shown certain limitations in uncovering the impact of *cis*-regulatory elements (CREs) on gene expression.[10,25–27]

Received 10 April 2025; accepted 20 August 2025;
https://doi.org/10.1016/j.omtn.2025.102696.

Correspondence: Guohua Wang, College of Computer and Control Engineering, Northeast Forestry University, Harbin 150040, China.
E-mail: ghwang@nefu.edu.cn

Published by Elsevier Inc. on behalf of The American Society of Gene and Cell Therapy.
This is an open access article under the CC BY license (http://creativecommons.org/licenses/by/4.0/).

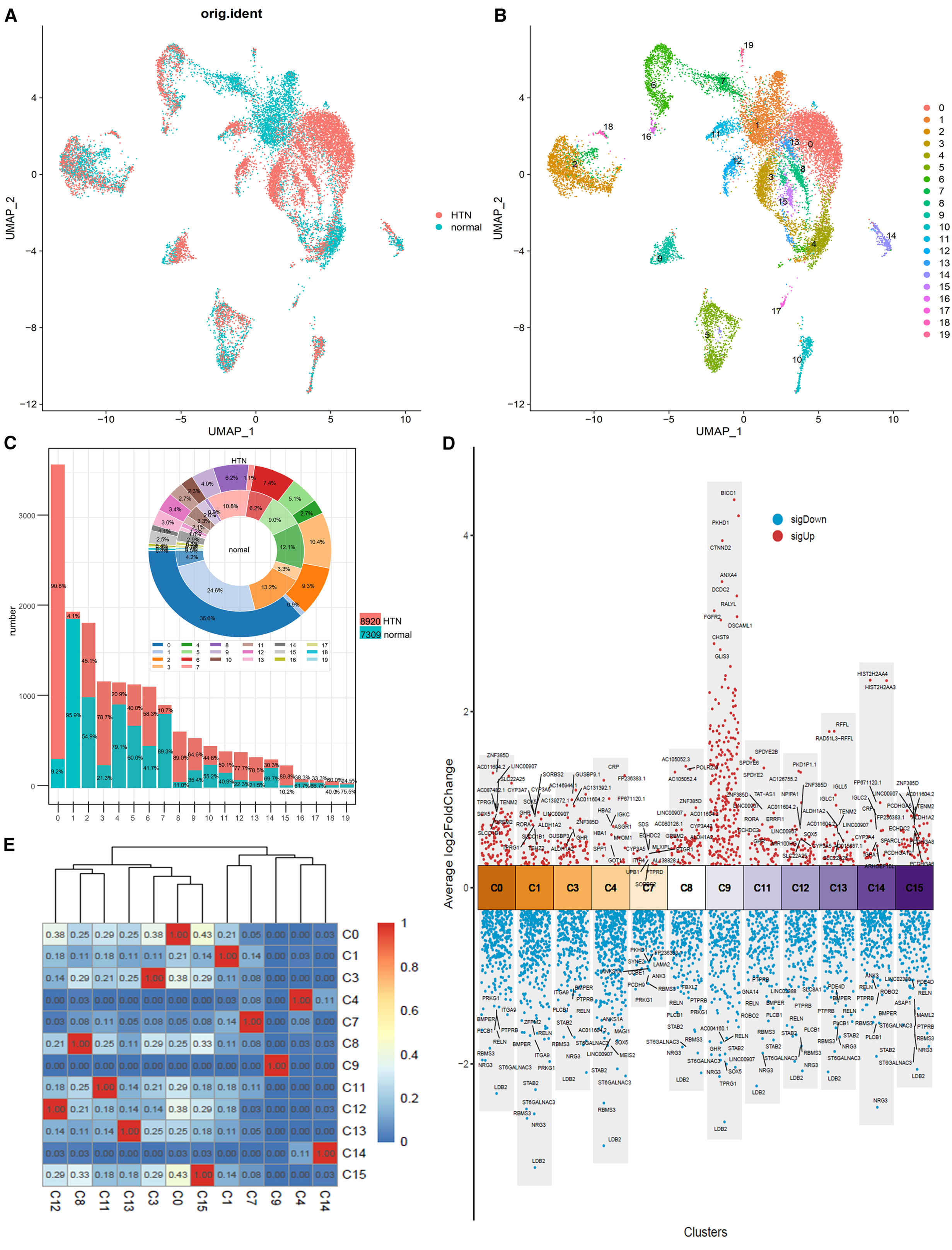

(legend on next page)

Potential disease-causing genes have been identified based on differentially expressed genes (DEGs) among cell types. However, the regulatory mechanisms of these genes have not received sufficient attention. The expression of DEGs is regulated by CREs, and understanding these CREs can help systematically and accurately infer the pathogenesis of diseases.[28–30] Single-cell assay for transposase-accessible chromatin using sequencing (scATAC-seq) can measure chromatin accessibility at single-cell resolution, identifying regions known as CREs that bind transcription factors (TFs) to control gene expression.[31–36] Multiple studies using scATAC-seq have demonstrated that CRE regulation promotes the high expression of pathogenic genes by recruiting TFs.[37]

Promoters and enhancers are the two major components of CREs previously reported to be associated with various diseases.[38–44] However, scATAC-seq alone cannot be used to directly distinguish between promoters and enhancers. To address this challenge, scATAC-seq needs to be combined with histone chromatin immunoprecipitation sequencing (ChIP-seq). Promoters enriched with H3K4me3 histone signals are typically located approximately 2,000 bp upstream of the transcription start site (TSS).[39,45] Enhancers, which are distal elements marked by H3K4me1 and H3K27ac histone signals, enhance gene transcription by recruiting TFs and acting in coordination with promoters.[46,47] Unfortunately, maps of promoters and enhancers at single-cell resolution are lacking, which makes it difficult to elucidate the regulatory networks of pathogenic genes in the context of disease occurrence and prevention.

In this study, we collected scRNA-seq, scATAC-seq, and histone ChIP-seq data from normal and HTN liver tissues. The scRNA-seq was used to explore the impact of HTN on the liver microenvironment by defining a novel HTN-associated liver cell subpopulation, termed Hepatocytes_1, and identifying five marker genes indicative of HTN. Subsequently, scATAC-seq was used to summarize the peak differences in Hepatocytes_1 cells and the distinct enrichment of TFs on these peaks. The peaks were annotated as promoters and enhancers based on histone ChIP-seq data. Furthermore, we investigated the specificity of TFs in Hepatocytes_1 cells under HTN and normal conditions. Finally, we constructed a gene-regulatory network (GRN) for Hepatocytes_1, providing evidence that the elevated expression of CYP3A4 was attributed to the binding of TFs to its promoter. Most importantly, the regulation of genes by promoters, enhancers, and TFs represents a potential trigger for HTN-induced liver disease.

RESULTS

Integration and mapping of normal and disease scRNA-seq

To investigate the gene and subpopulation disparities within liver tissues, scRNA-seq obtained from both normal and HTN liver tissues were integrated. A total of 8,920 HTN and 7,309 normal cells were segregated into 20 distinct cell clusters based on 2,000 highly variable genes using the Seurat package (Figures 1A and 1B). To identify the cell clusters associated with HTN, we conducted a comprehensive analysis by examining the distribution of normal and HTN cells within each cluster. Clusters 0, 1, and 2 accounted for the largest proportions of total cells. Notably, HTN cells in cluster 0 accounted for 90.8%, comprising 36.6% of all HTN cells. Conversely, cluster 1 showed a significantly higher proportion of normal cells compared to HTN cells (Table S1). The pronounced variation may provide insight into the cellular heterogeneity within liver cell subpopulations induced by HTN (Figures 1C and S1A).

DEGs are the fundamental drivers of cellular heterogeneity, showing substantial diversity between subtypes and homology within subtypes in terms of both the number of genes and their expression levels (Figures 1D, S1B, and S1C). Hence, 12 clusters were predicted to be hepatocytes using the SingleR[48] package (Figure 1D), whereas the remaining eight clusters were annotated as Kupffer cells, stellate cells (C6 and C16), T cells, B cells, endothelial cells (C2 and C18), and erythroblast cells (Figures S1C and S1D). In both hepatocyte clusters 0 and 1, the upregulated genes included SOX5, whereas LDB2 was downregulated in multiple hepatocyte clusters. However, there were significant differences in the upregulated genes between clusters 0 and 1. Furthermore, the expression differences of the upregulated genes in cluster 9 were notably greater than those in the other clusters (Figure 1D). This suggests that there is still heterogeneity within the hepatocyte subpopulations or the possibility of annotation errors.

To understand the heterogeneity within hepatocyte subpopulations, the top 20 upregulated genes (Equation 1) from 12 liver cell-related clusters were used to calculate the Jaccard similarity (Figure 1D; Table S2; Equation 2). Based on the hierarchical clustering results, the clusters were grouped into two distinct subtypes: C0, C3, C8, C11, C12, C13, and C15 were annotated as “Hepatocytes_1,” while C1, C4, C7, and C14 were annotated as “Hepatocytes_2.” Cluster C9 was not annotated as hepatocytes due to a Jaccard similarity of 0 with other cell clusters (Figures 1E and S1D). Interestingly, “Hepatocytes_1” clusters exhibited a higher abundance of HTN cells compared to normal cells, whereas “Hepatocytes_2” clusters contained more normal cells than HTN cells, highlighting the heterogeneity of hepatocyte subpopulations induced by HTN (Figures 1C, 1E, and S1D).

Cell annotation and identification of biological markers

The disparity in cell type proportions between the two hepatocyte subtypes reflects the impact of HTN on the liver cellular microenvironment. Therefore, we conducted a more rigorous manual annotation based on literature-validated cell marker genes.[18] As a result,

Figure 1. Integration of HTN and normal cells based on scRNA-seq

(A) and (B) UMAP of cell integration and clustering. (C) The proportion and number of cell clusters. (D) Differential genes in Hepatocytes_1-related clusters. (E) Hierarchical clustering heatmap of similarity for hepatocyte-related clusters based on the top 20 upregulated genes (avg_log2FC > 0 and p_val < 0.05).

all cell clusters were categorized into seven distinct cell subtypes: Hepatocytes_1, Hepatocytes_2, endothelial cells, Kupffer cells, stellate cells, cholangiocytes, and T cells (Figures 2A and S2A; Table S3). Notably, C9 was annotated as cholangiocytes, C17 as endothelial cells, and C19 as Hepatocytes_2. The marker genes showed high specificity within different subgroups. For example, LDB2 was highly expressed exclusively in endothelial cells (Figure 2B). Hepatocytes_1 and Hepatocytes_2 together constituted the primary cellular components of the liver, accounting for 40.9% and 26.7% of total liver cells, respectively. In Hepatocytes_1, HTN cells accounted for 85.3% of the total cells, while in Hepatocytes_2, normal cells accounted for the highest proportion (88%), suggesting that Hepatocytes_1 may play a crucial role in the development of HTN (Figure 2C; Table S4).

To elucidate the biological functions of each cell type, we analyzed the DEGs in each cell cluster and performed Gene Ontology functional enrichment analysis on the upregulated genes (Figure 2D; Table S5). The results revealed that Hepatocytes_1 was predominantly enriched in biological processes related to fatty acid metabolism and small-molecule catabolism (Figure 2E; Table S6), highlighting the close relationship between Hepatocytes_1 and HTN. These findings are consistent with the existing literature, which has documented the association between metabolic syndrome, nonalcoholic fatty liver disease, and altered hepatic metabolism, thus further supporting our observations.[4,5,25,49] Hepatocytes_2 showed enrichment in functions related to oxygen transport, which is consistent with the literature emphasizing the critical role of normal hepatocytes in oxygen and nutrient exchange.[25] T cells were enriched in pathways involved in T cell differentiation and activation, while endothelial cells were related to the regulation of vasculature development (Figure S2B). Collectively, these findings further validate the accuracy of our cell annotation results and highlight the complexity of the liver cellular microenvironment.

Gene markers of Hepatocytes_1 can serve as new therapeutic targets for HTN. We identified five key marker genes of Hepatocytes_1 that originated from the intersection of the upregulated genes in Hepatocytes_1 and the pathway-enriched genes (Figures 2F, 2G, and S2C; Table S7). UPB1 is an important enzyme involved in the pyrimidine degradation pathway,[50] and pyrimidine metabolism has been reported to be an important mechanism in the treatment of HTN.[1] SDS, primarily present in the liver, is responsible for the metabolism of serine and glycine, which are considered important biomarkers for the diagnosis of HTN.[51] Multiple studies have reported a direct connection between PCCA, CYP3A4, and PPARGC1A and the induction of HTN.[52–55] It is worth noting that CYP3A4 is involved in the metabolism of various drugs and lipids and serves as a substrate for the antihypertensive medication felodipine.[56] Additionally, the high expression of CYP3A4 reduces the resistance of HTN patients to oral administration of octreotide.[57] In summary, the overexpression of five marker genes in Hepatocytes_1 may contribute to HTN development, which could serve as a potential indicator for the occurrence of HTN and its associated liver diseases. Trajectory inference[58] indicates that normal Hepatocytes_1 cells are in an early differentiation phase compared to HTN cells, supporting gradual evolution of HTN-associated Hepatocytes_1 from a normal state to an HTN state (Figures 2H and S3A).

To further validate the trajectory inference results obtained from Monocle2, we employed scVelo analysis,[59] which leverages RNA velocity theory to reconstruct cell differentiation trajectories based on spliced and unspliced transcripts. Both methods consistently revealed that Hepatocytes_1_normal are positioned at the early stage of the trajectory, whereas HTN-associated Hepatocytes_1 progress toward a disease state (Figures S3B and S3D). Furthermore, the comparison of spliced and unspliced RNA proportions demonstrated distinct transcriptional states between these two states (Figure S3C). Hepatocytes_1_HTN exhibited a markedly higher proportion of unspliced RNA (71%) and a lower proportion of spliced RNA (22%), indicating enhanced transcriptional activity but decreased splicing efficiency. In contrast, Hepatocytes_1_normal had a higher proportion of spliced RNA (47%) and lower unspliced RNA (43%), reflecting a more balanced and mature transcriptional landscape. Together, these findings provide robust, multidimensional evidence that HTN induces significant changes in both the differentiation dynamics and gene expression regulation of Hepatocytes_1.

Cross-platform matching of scRNA-seq and scATAC-seq

Although HTN-associated cell subtypes and their marker genes have been defined and validated, gene-regulatory relationships at the single-cell resolution remain elusive. Detecting CREs and elucidating the regulatory rules using scATAC-seq are key steps to understanding the pathogenic mechanisms of HTN. The R package ArchR[60] categorized 40,652 HTN cells and 55,707 normal cells into 20 clusters for liver scATAC-seq (Figure 3A). Cell cluster C18, which has the largest proportion in scATAC-seq, primarily matches stellate cells in the scRNA-seq. Additionally, Hepatocytes_1 scRNA-seq matches scATAC-seq clusters C11 and C12 (Figure 3B). Then, the scATAC-seq clusters were annotated into six cell types based on scRNA-seq annotation results (Figure 3C). The "genescores" of marker genes for each cell cluster indicate that the annotation results of the scATAC-seq are trustworthy (Figure 3D).

Promoters play a crucial role in activating gene. Therefore, we investigated chromatin accessibility around the TSSs of the marker genes. The results revealed that, within Hepatocytes_1, marker genes of HTN state exhibited wider coverage of scATAC-seq fragments in the TSS flanking regions, leading to differences in gene expression. The potential promoter regions can easily interact with TFs and enhancers, promoting the high expression of marker genes in HTN cells (Figure 3E). For example, CYP3A4 has a more extended chromatin-accessible region upstream of its TSS in HTN than normal. CREs require binding with TFs to regulate gene expression. Therefore, the 163,166 peaks were called to explore potential CREs regions.

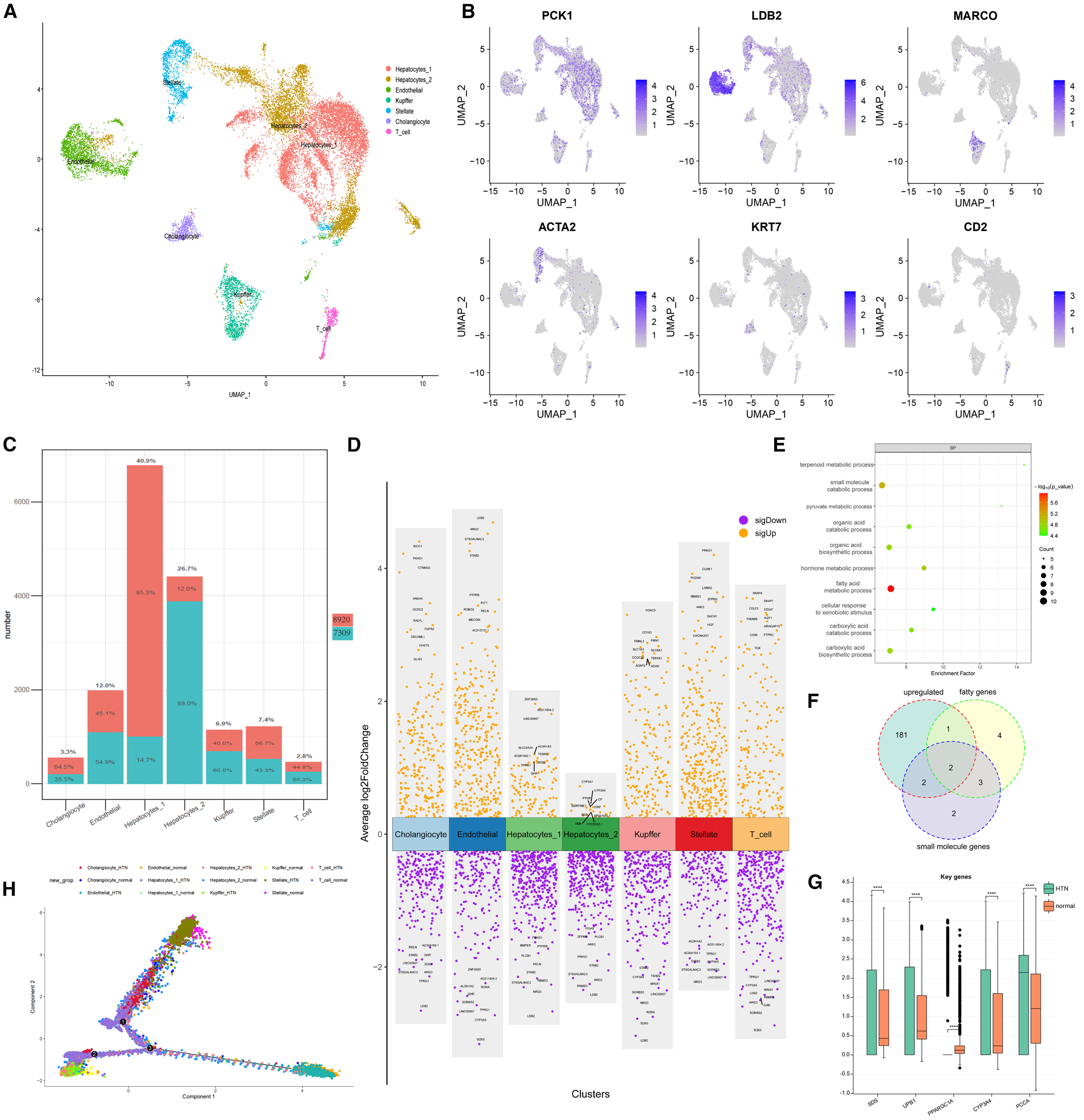

Figure 2. Cell annotation and heterogeneity analysis

(A) UMAP of annotated cell types. (B) Specific expression of cell marker genes. Hepatocyte: PCK1; endothelial: LDB2; Kupffer: MARCO; stellate: ACTA2; cholangiocyte: KRT7; T cell: CD2. (C) The proportion and number of HTN and normal cells across the seven cell types. (D) Differential genes among the cell types. (E) Functional enrichment analysis of Hepatocytes_1. (F) Venn diagram of HTN-related pathway genes (fatty acid metabolic process: 0006631 and small-molecule catabolic process: 0044282) and upregulated genes in Hepatocytes_1 (HTN cell vs. normal cell). (G) Differential expression of HTN-related marker genes in Hepatocytes_1 between HTN and normal cells. (H) Pseudo-time trajectories of 14 cell types, showing Hepatocytes_1 in the normal state differentiating earlier than in the HTN state.

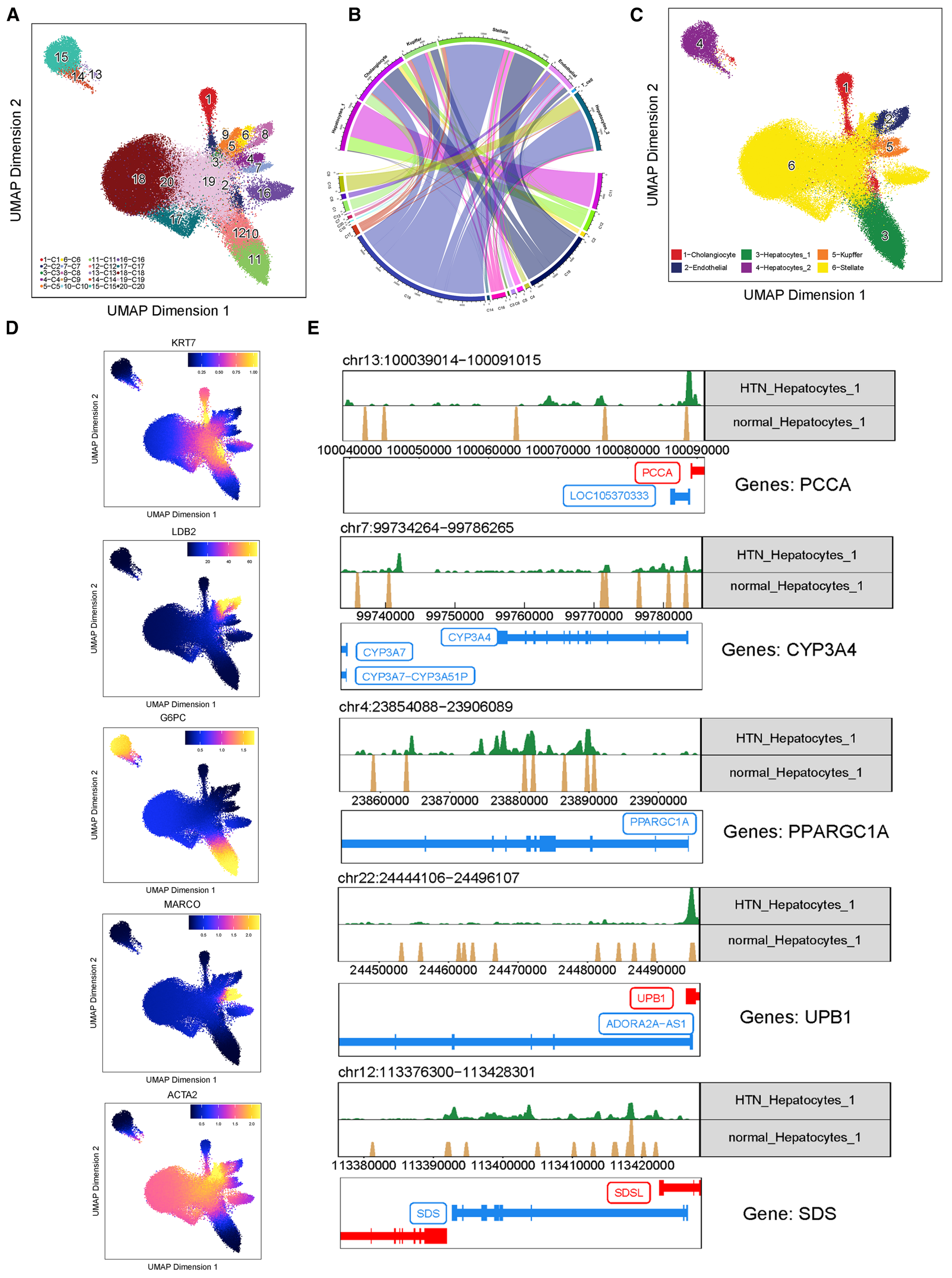

(legend on next page)

Complexity and diversity in peak distribution for Hepatocytes_1 cells

The quantity and location of peaks are directly associated with gene expression levels. A higher number of peaks implies more frequent interactions between TFs and CREs. Peaks located further from the TSS may function as enhancers, interacting with promoters within the TSS region to facilitate gene expression. ArchR categorizes peaks into four types based on their genomic positions: "Promoter," "Distal," "Exonic," and "Intronic." The distribution of the four types of peaks between the two states of Hepatocytes_1 exhibits a significant difference (Figure 4A; Table S8), as indicated by a chi-squared test (p value $<2.2 \times 10^{-16}$). The results indicate that "Intronic" peaks are the most numerous among the four types of peaks, with only a minor difference in number between HTN and normal Hepatocytes_1. "Exonic" peaks exhibit a quantity distribution trend similar to that of "Intronic" peaks. "Promoter" peaks in HTN cells account for 16.8%, whereas, in normal cells, they account for 8.3%. Interestingly, "Distal" peaks are less abundant in HTN cells compared to normal cells (Figure 4B). The opposing quantitative trends observed in the "Promoter" and "Distal" peaks may be the primary driving force behind the differential expression of marker genes.

Gene transcription levels correlate with the diversity of regulatory elements within a given region.[61–63] In HTN cells, the maximum number of both "Intronic" and "Distal" peaks within the same gene region reaches 146, and a similar trend is observed in normal cells. There are six genes covered by all four types of peaks in HTN cells, while no such genes are observed in normal cells (Figure 4C). Additionally, the number of genes covered by multiple peaks was significantly lower in normal cells than in HTN cells. Furthermore, the distribution of genes within each peak type was also investigated (Figure 4D). There are 362 common genes in the "Intronic" and 194 in the "Distal." However, "Promoter" and "Exonic" have only 19 and 13 common genes, respectively. This indicates that the diversity of peaks within specific gene regions is associated with the high expression of marker genes in HTN cells.

In the regions of the five marker genes, there is a significantly higher number of peaks in HTN compared to normal state of Hepatocytes_1, and "Promoter" peaks are consistently present near the TSS (Figure 4E). This result validated our hypothesis that the high expression of marker genes is attributed to differential regulation by CREs. There is a significant difference in the quantity of "Promoter" and "Distal" peaks, which may drive the transition of normal cells into the HTN state. Particularly, the absence of "Promoter" peaks in normal cells is a major reason for the high expression of marker genes in HTN cells.

Prediction of Hepatocytes_1 cell promoter and enhancer

The annotation of peaks by ArchR does not provide a precise definition of the specific biological functions of CREs. Open chromatin regions associated with active promoters and enhancers often undergo various histone modifications. To address this, 6,552 HTN peaks and 6,587 normal peaks of Hepatocytes_1 were overlapped with histone modification signals to gain insight into the functional roles of promoters and enhancers.

At the bulk level of liver, the H3K4me3 promoter and H3K4me1 enhancer signals were higher in HTN state than in normal state, whereas the H3K27ac enhancer signal showed minimal differences (Figure 5A). The overlap between Hepatocytes_1 scATAC-seq peaks and histone peaks shows that normal cells have the lowest coverage of the H3K4me1 signal, suggesting that enhancers in normal Hepatocytes_1 are inactive. The H3K4me3 signal showed the smallest difference between HTN and normal cells when matched with scATAC-seq peaks, relative H3K4me1 and H3K27ac. However, significant differences still existed, as evidenced by the proportion of peaks not matched in HTN being 56.68% and in normal being 85.20%, indicating heterogeneity of CREs in the Hepatocytes_1 cell (Figure 5B).

To gain a more accurate understanding of the regulatory functions of promoters and enhancers on genes, stricter peak annotation was employed. "Promoter" peaks with H3K4me3 coverage are predicted as promoters, while "Distal," "Exonic," and "Intronic" peaks covered by both H3K4me1 and H3K27ac are predicted as enhancers. In the HTN state, we predicted 983 promoters and 1,642 enhancers (Tables S9 and S10), whereas, in the normal state, we predicted 396 promoters and 44 enhancers (Figures 5C and S4A; Tables S11 and S12). In HTN cells, both promoters and enhancers exhibited a clear bimodal distribution of histone signals, confirming the accuracy of our identification (Figure 5D). The disruption of the enhancer peaks observed in normal samples can be attributed to the inactive H3k4me1 signal in normal bulk cells, although some distinct peaks were still present (Figure S4B). The biological functions of promoters and enhancers rely on TF binding. The investigation of TF motif enrichment in promoter and enhancer regions revealed a high degree of cell specificity between normal and HTN state (Figure 5E). For example, TBX21 is specifically enriched in enhancers of HTN cells and is involved in liver inflammatory responses and immune cell activation.[64] The cell-specific regulation of promoters and enhancers, mediated by TFs, is the main reason for the high expression of the marker gene.

The regulatory networks of upregulated genes for Hepatocytes_1 in the HTN state

GRNs derived solely from scRNA-seq data lack the cell specificity provided by CREs and TFs. Therefore, the GRNs of Hepatocytes_1

Figure 3. Cell matching between scRNA-seq and scATAC-seq

(A) UMAP clustering of scATAC-seq. (B) Matching of scATAC-seq and scRNA-seq cells based on RNA expression levels and gene scores. (C) UMAP of scATAC-seq cell types based on scRNA-seq annotations. (D) UMAP of marker gene scores for five cell types of scATAC-seq. Cholangiocyte: KRT7; endothelial: LDB2; hepatocyte (cluster 3 and 4): G6PC; Kupffer: MARCO; stellate: ACTA2. (E) Coverage of scATAC-seq fragments upstream of the TSS for Hepatocytes_1 marker genes.

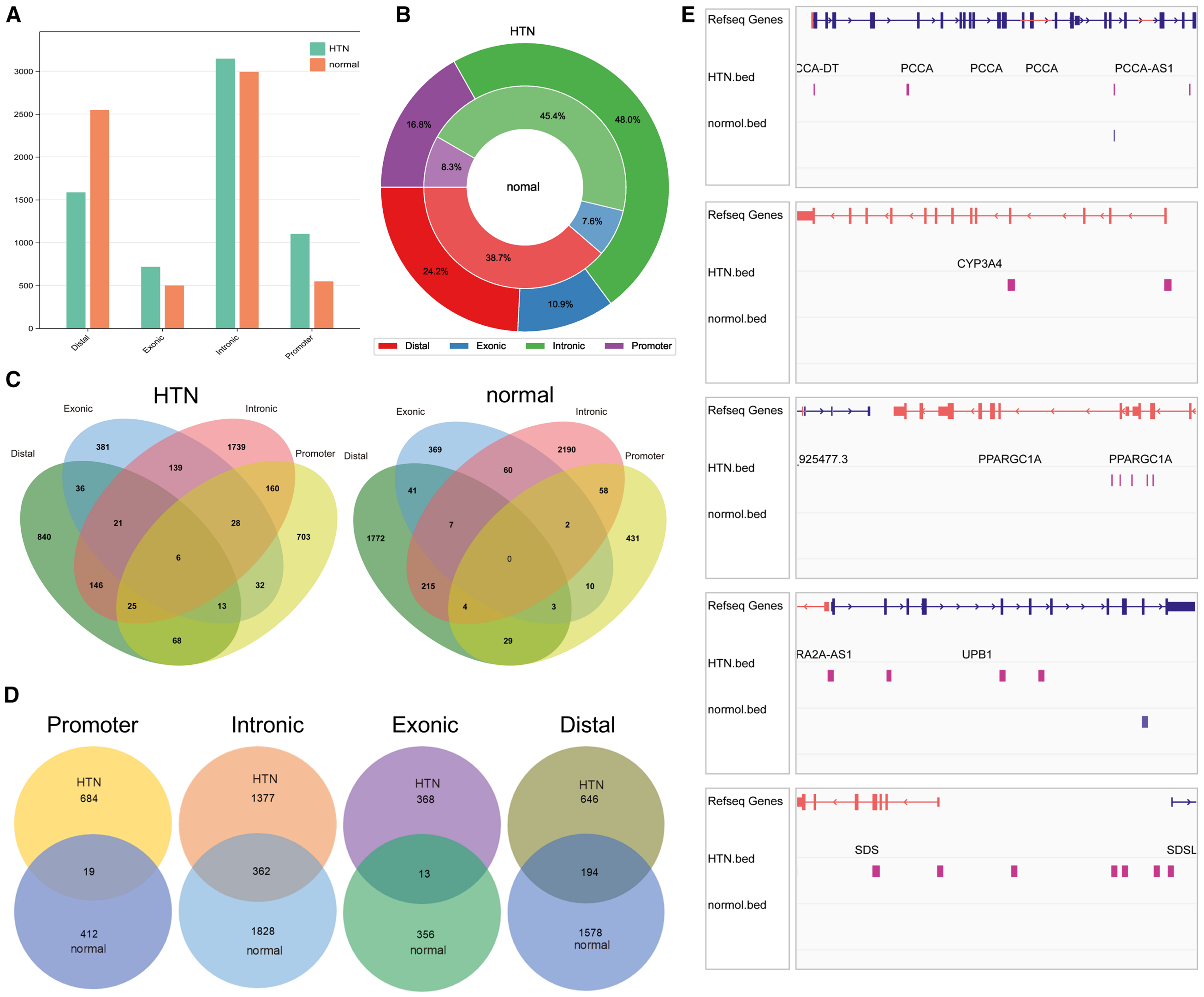

Figure 4. Heterogeneity in peak profiles between normal and HTN-Hepatocytes_1

(A) The number of each of the four types of peaks. (B) The proportion of peaks in normal and HTN-Hepatocytes_1 cells. (C) Venn diagrams showing shared and distinct gene regions among the four types of peaks within the same cell state. (D) Venn diagram showing shared and distinct gene regions of peaks between HTN and normal states of Hepatocytes_1. (E) The distribution of peaks in marker genes.

in HTN state were constructed based on the interactions between TFs and CREs by integrating scATAC-seq and histone ChIP-seq.

Among the 983 predicted target genes of the promoters, six genes are upregulated in the HTN state compared to normal Hepatocytes_1 (Figure 6A; Table S13). Notably, we inferred the regulatory relationship of CYP3A4 in HTN cells, whose promoter is enriched with motifs for five TFs (Figure 6B). The absence of the promoter in normal cells resulted in low expression of CYP3A4. The network includes 16 TFs, all of which are part of the 2,000 DEGs. TF WT1 is associated with liver cirrhosis[65] and linked to four genes. GATA4 helps form small blood vessels, and lacking it might cause high blood pressure and liver problems.[66] The regulation of gene expressions by enhancers and TFs is more complex, with a single gene potentially having multiple enhancers. Therefore, a stringent enhancer-mediated regulatory network was constructed based on SCENIC+,[67] GeneHancer,[68] and histone ChIP-seq data (Figures 6C and 6D). The UPB1 gene encodes β-ureidopropionase, an enzyme involved in the pyrimidine degradation pathway and closely associated with liver metabolic status.[69] NR1H4 (also known as farnesoid X receptor) is a key TF for maintaining hepatic metabolic homeostasis, regulating bile acid metabolism and hepatocyte function.[50,70] Enhancer-mediated regulatory network analysis indicates that NR1H4 regulates UPB1 expression via the enhancer located at

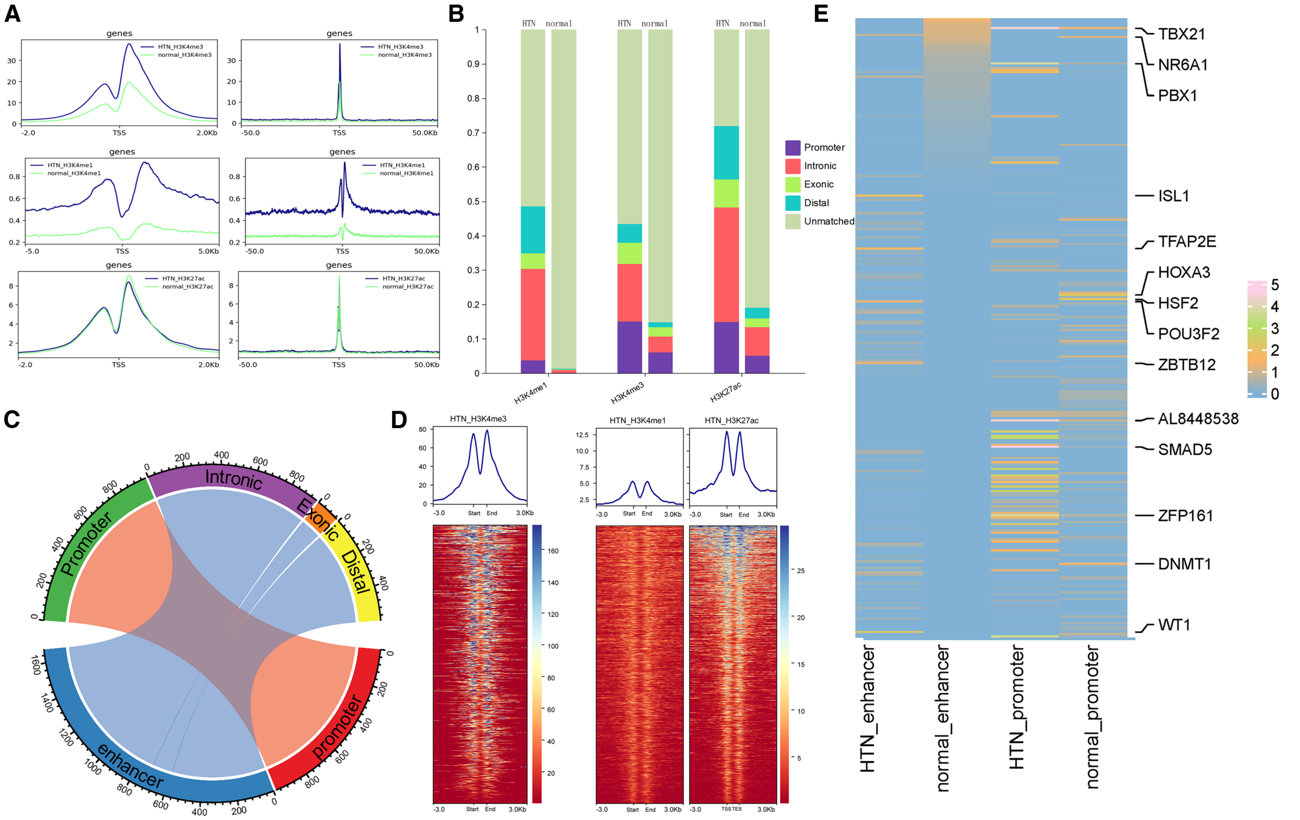

Figure 5. The annotation of peaks is based on histone signals

(A) Bulk-level histone signals in normal and HTN liver tissues. (B) Overlap between histone signals and peaks in Hepatocytes_1. (C) Annotation of promoter and enhancer regions for HTN-specific peaks in Hepatocytes_1. (D) Distribution of histone signals in promoter and enhancer regions of Hepatocytes_1 HTN cells. (E) Enrichment of TFs in the promoter and enhancer regions of Hepatocytes_1.

chr22:24510675-24511175. The potential regulatory relationship promotes the upregulation of UPB1, which may help alleviate liver metabolic disturbances induced by HTN.

The aforementioned regulatory mechanisms provide novel insights into the pathogenesis of HTN and suggest that HTN may contribute to the development of liver-related diseases, thereby opening new avenues for therapeutic interventions targeting both HTN and associated hepatic conditions. For instance, cell-specific promoters or enhancer sequences can be artificially synthesized,[71,72] or CRISPR-mediated editing of CREs[73,74] can be employed to modulate TF binding patterns. Such strategies could facilitate the correction of pathological GRNs and ultimately inhibit the progression of HTN and its related liver disease.

DISCUSSION

The development of single-cell sequencing technology has provided new therapeutic approaches for complex diseases such as cancer through the discovery of marker genes. However, significant gaps remain in the understanding of chronic diseases and CREs. In the present study, we conducted a comprehensive investigation of the impact of HTN on the liver tissue microenvironment based on multi-omics data, which encompassed aspects such as cellular heterogeneity, DEGs, annotation of CREs, and construction of GRNs.

We observed a highly intriguing cellular heterogeneity in liver cells, where HTN cells in Hepatocytes_1 constituted the majority (85.3%), while Hepatocytes_2 had a predominance of normal cells, suggesting that the patient's long history of HTN led to significant differentiation in hepatocyte populations. Functional enrichment and DEGs provided compelling evidence indicating that Hepatocytes_1 is most affected by HTN. Within this cell type, we have successfully identified five marker genes that have been confirmed to be closely associated with the onset of HTN. Trajectory inference also revealed that normal cells in Hepatocytes_1 were at an earlier differentiation stage than HTN cells, implying that the state and marker genes of Hepatocytes_1 may play a role in the prevention and treatment of HTN.

The application of scRNA-seq often limits the exploration of the regulatory mechanisms of disease marker genes. Considering the dominant influence of CREs on gene expression, we investigated

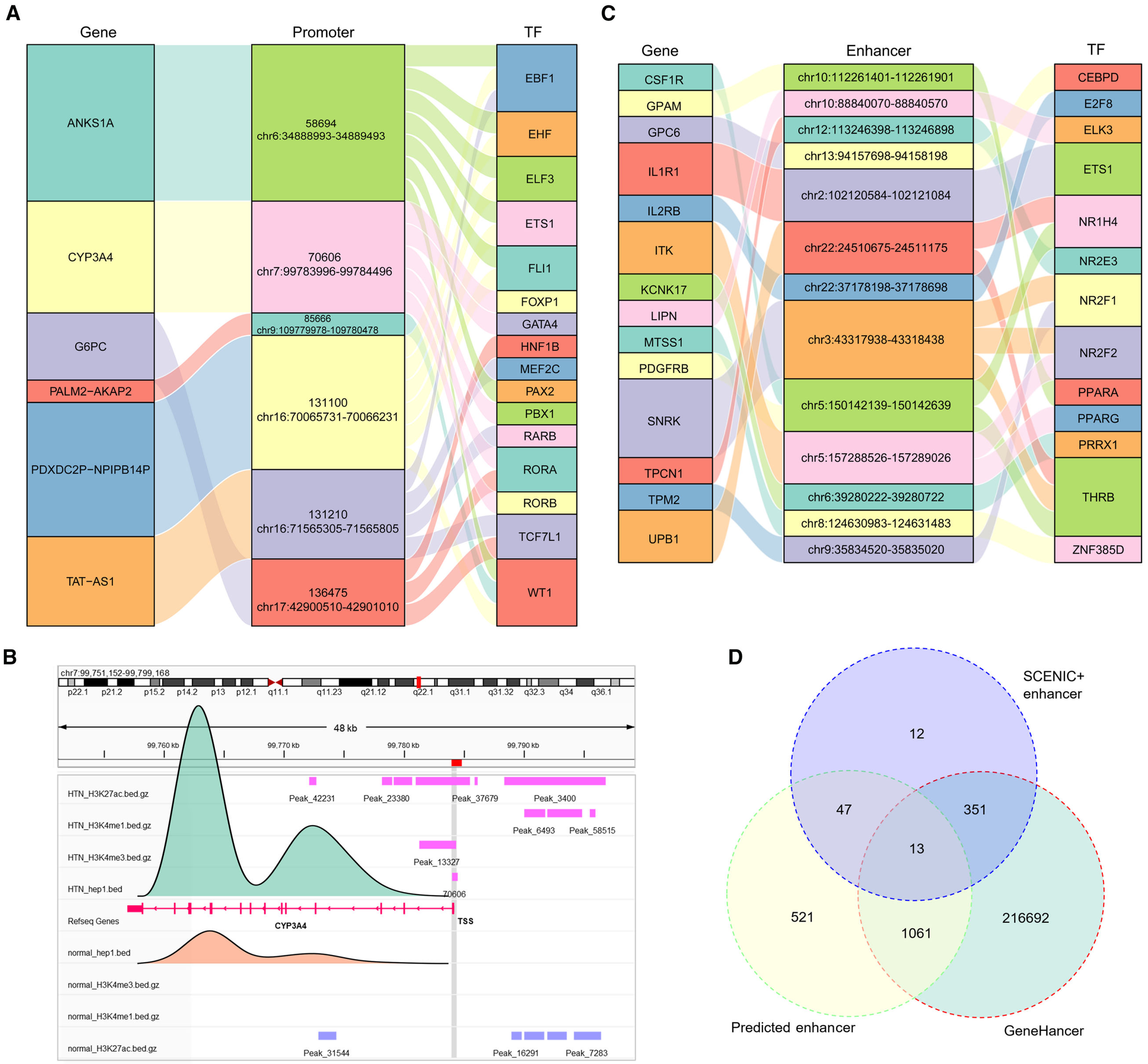

Figure 6. Regulatory network of upregulated genes in Hepatocytes_1 under HTN condition

(A) The regulatory network for upregulated genes with promoters. (B) The distribution of histone signals near the TSS of CYP3A4 in normal and HTN cells. The peak plots in light green and light orange represent the gene expression of CYP3A4 in HTN and normal cells, respectively. (C) The regulatory network of genes associated with predicted enhancers that are shared by both SCENIC+ and GenHancer. (D) Venn diagram showing the overlap of predicted enhancers identified by histone ChIP-seq, SCENIC+ inference, and GenHancer annotation.

the regulatory mechanisms of biological markers with a focus on the regulatory roles of promoters and enhancers of gene expression. The variation in peak distribution in Hepatocytes_1 has drawn significant attention based on scATAC-seq. There is a significant difference in the quantity of "Promoter" and "Distal" peaks, leading to differentiation between HTN and normal cells, especially in the absence of "Promoter" peaks for all normal cells, particularly in the case of marker genes. Histone ChIP-seq provides an opportunity to explore the regulatory rules of promoters and enhancers of TF-mediated gene expression. The TFs enriched in promoters and enhancers exhibited cell specificity. In the regulatory network of Hepatocytes_1, the regulatory mechanism of CYP3A4 may be involved in the differentiation of normal cells into HTN cells. The upregulation of gene expression in the HTN state is regulated by CREs and TFs, which implies the damaging effects of HTN on liver health.

However, in this study, the scRNA and scATAC data were generated using single-molecule single-cell sequencing technologies, and there were no shared cellular barcodes linking the different cells. As a result, bioinformatics tools were required to match the omics data between cells, which may lead to potential biases in the experimental results. To date, single-cell multi-omics sequencing data with shared cellular identities have become more mature and reliable. In the future, applying these technologies to investigate the impact of HTN on liver health is expected to yield more accurate and higher-resolution biological markers.

Conclusions

HTN enhances the heterogeneity of the liver microenvironment, and the newly defined Hepatocytes_1 play a role in preventing and monitoring the progression of HTN. The five marker genes identified in Hepatocytes_1 can serve as therapeutic targets for HTN and its associated liver complications. The regulatory mechanisms of promoters and enhancers explain the differential expression levels of marker genes and illustrate that specific genes or TFs are key factors in HTN-induced liver disease.

MATERIALS AND METHODS

Cell clustering and integrating for scRNA-seq

Seurat (version 4.3.0) was used for integration and clustering of scRNA-seq data derived from liver tissues under both normal and HTN condition.[75] After integration, the standard Seurat pipeline was employed, including quality control, normalization ("NormalizeData"), highly variable gene identification ("FindVariableFeatures," with vst method and 2,000 genes retained), and dimensionality reduction ("RunPCA," npcs = 30; "RunUMAP," dims = 1:30). The "FindNeighbors" and "FindClusters" functions were used for cell clustering with a resolution parameter set to 0.5, resulting in 20 clusters. This resolution was chosen according to Seurat guidelines to ensure appropriate segmentation and to adequately capture cell diversity; the resulting clusters were confirmed to be biologically meaningful through marker gene analysis and subsequent cell type annotation. DEGs within the clusters were identified using the "FindAllMarkers" function, and these DEGs were used for downstream cell annotation and functional enrichment analysis.

Cell annotation and determination of hepatocyte subtypes

SingleR (version 2.0.0) was used for the initial annotation of the cell clusters. The Jaccard index was employed to assess hepatocyte similarity using the top 20 DEGs sorted based on avg_log2FC and annotated as hepatocyte-related clusters.

$$c_i = \{gene\ name_1, gene\ name_2, \ldots, gene\ name_{20}\} \quad \text{(Equation 1)}$$

$$J(c_i, c_j) = \frac{|c_i \cap c_j|}{|c_i \cup c_j|}, c_i, c_j \in Hepatocytes \quad \text{(Equation 2)}$$

The results of hierarchical clustering ("pheatmap" with default parameters) divided hepatocytes into two cell subtypes based on similarity. Finally, manual corrections were made to the cell annotations based on cell gene markers found in the literature.

Functional enrichment

The functional enrichment of cell subpopulations was assessed using the "enrichGO" function, and the "findMarkers" function was used to obtain a gene list for annotating the biological processes of the cells.

Pseudo-time analysis

The pseudo-time trajectory was constructed using the R package monocle, and manually annotated scRNA-seq cell clusters were mapped onto the differentiation trajectory. scVelo was employed to assess the reliability of Monocle2 trajectory inference.

Matching of scRNA-seq and scATAC-seq

Cell annotation in the scATAC-seq relied on scRNA-seq, and the ArchR package was used to match the scRNA-seq and scATAC-seq cells. Similar to the scRNA-seq processing pipeline, ArchR performs standard operations on scATAC-seq, including quality control, dimensionality reduction, and clustering. Subsequently, scATAC-seq clusters and scRNA-seq clusters were matched using the "addGeneIntegrationMatrix" function, which was rigorously performed based on the background of HTN cell IDs and normal cell IDs.

Calling peaks and motif enrichment

To identify open chromatin regions, we performed peak calling on the scATAC-seq fragments using the "addReproduciblePeakSet" function in the ArchR package (v.1.0.2), which internally utilizes the MACS2 algorithm. MACS2 is a widely used tool designed to identify regions of significant enrichment in both ATAC-seq and ChIP-seq data by modeling the distribution of sequencing reads to detect peaks that represent enriched chromatin-accessible regions. In this study, MACS2 was run with the following parameters: genome size of 2.7×10^9, input format as bed, calling peak summits, retaining all duplicate reads, no model built, no local lambda correction, shifting fragment ends by −75 bp, extension size of 150 bp, and a q value threshold of 0.1. Subsequently, the "addMotifAnnotations" function was used to enrich and annotate TF motifs in the identified peaks.

Prediction of promoters and enhancers

The peaks annotated as "Promoter" by ArchR and overlapping with H3K4me3 regions are determined as promoters. Peaks annotated as other types by ArchR and overlapping with both H3K4me1 and H3K27ac regions were designated as enhancers (Figure S5). Overlapping regions were calculated using bedtools.[76] Histone signals in the promoter and enhancer regions were verified using deepTools.[77]

The construction of the regulatory network

In the Hepatocytes_1 promoter regulatory network under HTN conditions, the selection of genes is based on those upregulated in Hepatocytes_1 (avg_log2FC > 0 and p_val < 0.05) whose promoter regions

overlap with H3K4me3. The initial construction of the enhancer-mediated regulatory network was based on pseudo-multiomic data inferred by SCENIC+ from scRNA-seq and scATAC-seq. Regulatory relationships between enhancers and genes that were not supported by GenHancer annotations were subsequently removed. In addition, enhancer regions lacking overlap with both H3K4me1 and H3K27ac marks were excluded. Consequently, a stringent regulatory network meeting all of these criteria was ultimately constructed.

DATA AVAILABILITY

The normal multi-omics data for liver tissue were obtained from ENCODE.[78,79] https://www.encodeproject.org/biosamples/ENCBS790AKV/. The pre-processed scRNA-seq gene count matrix is available at ENCFF707EOQ, and the scATAC-seq fragment data at ENCFF289FJG. The histone ChIP-seq data in bed narrowPeak format for H3K4me1, H3K4me3, and H3K27ac were downloaded from ENCFF608RNK, ENCFF830CCA, and ENCFF837ELM. The HTN multi-omics data were obtained at https://www.encodeproject.org/biosamples/ENCBS344HUA/, including pre-processed scRNA count matrix from ENCFF429WMQ and the scATAC-seq fragments from ENCFF240WBA. The histone ChIP-seq data for H3K4me1, H3K4me3, and H3K27ac can be found at: https://www.encodeproject.org/biosamples/ENCBS558OUC/, the processed bed narrowPeak files were downloaded at ENCFF189UID, ENCFF504QPL, and ENCFF271HJI. Custom code used for this paper is available from GitHub at https://github.com/Hongfeipower/HTN.

ACKNOWLEDGMENTS

This work has been partially supported by the National Key Research and Development Program of China (2024YFE0213800 and 2022YFF1202101) and the National Natural Science Foundation of China (62131004, 62302342, 62225109, and 62072095).

AUTHOR CONTRIBUTIONS

Conceptualization, H. Li, M.Z., H. Lin, and G.W.; methodology, L.C., Y.Z., and Y.L.; investigation, H. Li, A.W.C.K., and H. Lin; writing – original draft, H. Li; writing – review and editing, H. Li and G.W.; funding acquisition, M.Z.; resources, Y.L.; validation, Q.Z. and G.W.; supervision, G.W.; project administration, Q.Z.

DECLARATION OF INTERESTS

The authors declare no competing interests.

SUPPLEMENTAL INFORMATION

Supplemental information can be found online at https://doi.org/10.1016/j.omtn.2025.102696.

REFERENCES

1. Yu, Z., Zhou, M., Liu, J., and Zhao, W. (2022). Underlying antihypertensive mechanism of egg white-derived peptide QIGLF using renal metabolomics analysis. Food Res. Int. *157*, 111457. https://doi.org/10.1016/j.foodres.2022.111457.
2. Dzau, V.J., and Hodgkinson, C.P. (2024). Precision Hypertension. Hypertension *81*, 702–708. https://doi.org/10.1161/HYPERTENSIONAHA.123.21710.
3. Al-Makki, A., DiPette, D., Whelton, P.K., Murad, M.H., Mustafa, R.A., Acharya, S., Beheiry, H.M., Champagne, B., Connell, K., Cooney, M.T., et al. (2022). Hypertension Pharmacological Treatment in Adults: A World Health Organization Guideline Executive Summary. Hypertension *79*, 293–301. https://doi.org/10.1161/hypertensionaha.121.18192.
4. Moller, S., and Bernardi, M. (2013). Interactions of the heart and the liver. Eur. Heart J. *34*, 2804. https://doi.org/10.1093/eurheartj/eht246.
5. Lonardo, A., Nascimbeni, F., Mantovani, A., and Targher, G. (2018). Hypertension, diabetes, atherosclerosis and NASH: Cause or consequence? J. Hepatol. *68*, 335–352. https://doi.org/10.1016/j.jhep.2017.09.021.
6. López-Suárez, A., Guerrero, J.M.R., Elvira-González, J., Beltrán-Robles, M., Cañas-Hormigo, F., and Bascuñana-Quirell, A. (2011). Nonalcoholic fatty liver disease is associated with blood pressure in hypertensive and nonhypertensive individuals from the general population with normal levels of alanine aminotransferase. Eur. J. Gastroenterol. Hepatol. *23*, 1011–1017. https://doi.org/10.1097/MEG.0b013e32834b8d52.
7. Danpanichkul, P., Ng, C.H., Muthiah, M.D., Duangsonk, K., Kongarin, S., Srisurapanont, K., Pingwang, P., Songmueang, N., Nonthasoot, C., Manosroi, W., et al. (2024). Metabolic Syndrome and Metabolic Dysfunction-Associated Steatotic Liver Disease in Premenopausal Women: Global Trends and Projections to 2040. Mayo Clin. Proc. *99*, 1615–1628. https://doi.org/10.1016/j.mayocp.2023.12.025.
8. Danpanichkul, P., Suparan, K., Dutta, P., Kaeosri, C., Sukphutanan, B., Pang, Y., Kulthamrongsri, N., Jaisa-aad, M., Ng, C.H., Teng, M., et al. (2024). Disparities in metabolic dysfunction-associated steatotic liver disease and cardiometabolic conditions in low and lower middle-income countries: a systematic analysis from the global burden of disease study 2019. Metabolism *158*, 155958. https://doi.org/10.1016/j.metabol.2024.155958.
9. Cui, L., Guo, G., Ng, M.K., Zou, Q., and Qiu, Y. (2024). GSTRPCA: irregular tensor singular value decomposition for single-cell multi-omics data clustering. Brief. Bioinform. *26*, bbae649. https://doi.org/10.1093/bib/bbae649.
10. Vandereyken, K., Sifrim, A., Thienpont, B., and Voet, T. (2023). Methods and applications for single-cell and spatial multi-omics. Nat. Rev. Genet. *24*, 494–515. https://doi.org/10.1038/s41576-023-00580-2.
11. Ma, R.-Y., Black, A., and Qian, B.-Z. (2022). Macrophage diversity in cancer revisited in the era of single-cell omics. Trends Immunol. *43*, 546–563. https://doi.org/10.1016/j.it.2022.04.008.
12. Lotfollahi, M., Rybakov, S., Hrovatin, K., Hediyeh-zadeh, S., Talavera-López, C., Misharin, A.V., and Theis, F.J. (2023). Biologically informed deep learning to query gene programs in single-cell atlases. Nat. Cell Biol. *25*, 337–350. https://doi.org/10.1038/s41556-022-01072-x.
13. Brunner, A.-D., Thielert, M., Vasilopoulou, C., Ammar, C., Coscia, F., Mund, A., Hoerning, O.B., Bache, N., Apalategui, A., Lubeck, M., et al. (2022). Ultra-high sensitivity mass spectrometry quantifies single-cell proteome changes upon perturbation. Mol. Syst. Biol. *18*, e10798. https://doi.org/10.15252/msb.202110798.
14. Zheng, S., Liang, J.-Y., Tang, Y., Xie, J., Zou, Y., Yang, A., Shao, N., Kuang, X., Ji, F., Liu, X., et al. (2023). Dissecting the role of cancer-associated fibroblast-derived biglycan as a potential therapeutic target in immunotherapy resistance: A tumor bulk and single-cell transcriptomic study. Clin. Transl. Med. *13*, e1189. https://doi.org/10.1002/ctm2.1189.
15. Lavie, D., Ben-Shmuel, A., Erez, N., and Scherz-Shouval, R. (2022). Cancer-associated fibroblasts in the single-cell era. Nat. Cancer *3*, 793–807. https://doi.org/10.1038/s43018-022-00411-z.
16. Kumar, V., Ramnarayanan, K., Sundar, R., Padmanabhan, N., Srivastava, S., Koiwa, M., Yasuda, T., Koh, V., Huang, K.K., Tay, S.T., et al. (2022). Single-Cell Atlas of Lineage States, Tumor Microenvironment, and Subtype-Specific Expression Programs in Gastric Cancer. Cancer Discov. *12*, 670–691.
17. Gambardella, G., Viscido, G., Tumaini, B., Isacchi, A., Bosotti, R., and di Bernardo, D. (2022). A single-cell analysis of breast cancer cell lines to study tumour heterogeneity and drug response. Nat. Commun. *13*, 1714. https://doi.org/10.1038/s41467-022-29358-6.
18. Wesley, B.T., Ross, A.D.B., Muraro, D., Miao, Z., Saxton, S., Tomaz, R.A., Morell, C.M., Ridley, K., Zacharis, E.D., Petrus-Reurer, S., et al. (2022). Single-cell atlas of human liver development reveals pathways directing hepatic cell fates. Nat. Cell Biol. *24*, 1487–1498. https://doi.org/10.1038/s41556-022-00989-7.
19. Wang, Z., Ding, H., and Zou, Q. (2020). Identifying cell types to interpret scRNA-seq data: how, why and more possibilities. Brief. Funct. Genomics *19*, 286–291. https://doi.org/10.1093/bfgp/elaa003.
20. Qi, R., Ma, A., Ma, Q., and Zou, Q. (2020). Clustering and classification methods for single-cell RNA-sequencing data. Brief. Bioinform. *21*, 1196–1208. https://doi.org/10.1093/bib/bbz062.
21. Li, T., Wang, Z., Liu, Y., He, S., Zou, Q., and Zhang, Y. (2025). An overview of computational methods in single-cell transcriptomic cell type annotation. Brief. Bioinform. *26*, bbaf207. https://doi.org/10.1093/bib/bbaf207.
22. Tan, D., Wang, J., Cheng, Z., Su, Y., and Zheng, C. (2024). Inferring Gene Regulatory Networks from Single-Cell Time-Course Data Based on Temporal Convolutional

Networks. Curr. Bioinform. *19*, 752–764. https://doi.org/10.2174/0115748936282613231211112920.

23. Zhu, Y., Bai, L., Ning, Z., Fu, W., Liu, J., Jiang, L., Fei, S., Gong, S., Lu, L., Deng, M., and Yi, M. (2024). Deep Learning for Clustering Single-cell RNA-seq Data. Curr. Bioinform. *19*, 193–210. https://doi.org/10.2174/1574893618666221130094050.

24. McConnell, M.J., Kostallari, E., Ibrahim, S.H., and Iwakiri, Y. (2023). The evolving role of liver sinusoidal endothelial cells in liver health and disease. Hepatology *78*, 649–669. https://doi.org/10.1097/hep.0000000000000207.

25. Ramachandran, P., Matchett, K.P., Dobie, R., Wilson-Kanamori, J.R., and Henderson, N.C. (2020). Single-cell technologies in hepatology: new insights into liver biology and disease pathogenesis. Nat. Rev. Gastroenterol. Hepatol. *17*, 457–472. https://doi.org/10.1038/s41575-020-0304-x.

26. Metwaly, A., Reitmeier, S., and Haller, D. (2022). Microbiome risk profiles as biomarkers for inflammatory and metabolic disorders. Nat. Rev. Gastroenterol. Hepatol. *19*, 383–397. https://doi.org/10.1038/s41575-022-00581-2.

27. He, X., Liu, X., Zuo, F., Shi, H., and Jing, J. (2023). Artificial intelligence-based multi-omics analysis fuels cancer precision medicine. Semin. Cancer Biol. *88*, 187–200. https://doi.org/10.1016/j.semcancer.2022.12.009.

28. Yadav, D.K., Shrestha, S., Dadhwal, G., and Chandak, G.R. (2018). Identification and characterization of cis-regulatory elements insulator and repressor' in *PPARD* gene. Epigenomics *10*, 613–627. https://doi.org/10.2217/epi-2017-0139.

29. Mathelier, A., Shi, W., and Wasserman, W.W. (2015). Identification of altered *cis*-regulatory elements in human disease. Trends Genet. *31*, 67–76. https://doi.org/10.1016/j.tig.2014.12.003.

30. Cherry, T.J., Yang, M.G., Harmin, D.A., Tao, P., Timms, A.E., Bauwens, M., Allikmets, R., Jones, E.M., Chen, R., De Baere, E., and Greenberg, M.E. (2020). Mapping the *cis*-regulatory architecture of the human retina reveals noncoding genetic variation in disease. Proc. Natl. Acad. Sci. USA *117*, 9001–9012. https://doi.org/10.1073/pnas.1922501117.

31. Ing-Simmons, E., Vaid, R., Bing, X.Y., Levine, M., Mannervik, M., and Vaquerizas, J. M. (2021). Independence of chromatin conformation and gene regulation during Drosophila dorsoventral patterning. Nat. Genet. *53*, 487–499. https://doi.org/10.1038/s41588-021-00799-x.

32. Stuart, T., Srivastava, A., Madad, S., Lareau, C.A., and Satija, R. (2021). Single-cell chromatin state analysis with Signac. Nat. Methods *18*, 1333–1341. https://doi.org/10.1038/s41592-021-01282-5.

33. Chen, M. (2025). Capturing cell-type-specific activities of cis-regulatory elements from peak-based single-cell ATAC-seq. Cell Genom. *5*, 100806. https://doi.org/10.1016/j.xgen.2025.100806.

34. Wang, D., Hu, X., Ye, H., Wang, Y., Yang, Q., Liang, X., Wang, Z., Zhou, Y., Wen, M., Yuan, X., et al. (2023). Cell-specific clock-controlled gene expression program regulates rhythmic fiber cell growth in cotton. Genome Biol. *24*, 49. https://doi.org/10.1186/s13059-023-02886-0.

35. Wei, J., Resztak, J.A., Ranjbaran, A., Alazizi, A., Mair-Meijers, H.E., Slatcher, R.B., Zilioli, S., Wen, X., Luca, F., Pique-Regi, R., et al. (2025). Functional characterization of eQTLs and asthma risk loci with scATAC-seq across immune cell types and contexts. Am. J. Hum. Genet. *112*, 301–317. https://doi.org/10.1016/j.ajhg.2024.12.017.

36. Wang, Y., Zhai, Y., Ding, Y., and Zou, Q. (2024). SBSM-Pro: support bio-sequence machine for proteins. Sci. China Inf. Sci. *67*, 212106. https://doi.org/10.1007/s11432-024-4171-9.

37. Wang, T., Shao, W., Huang, Z., Tang, H., Zhang, J., Ding, Z., and Huang, K. (2021). MOGONET integrates multi-omics data using graph convolutional networks allowing patient classification and biomarker identification. Nat. Commun. *12*, 3445. https://doi.org/10.1038/s41467-021-23774-w.

38. Li, M., Paik, H.I.H., Balch, C., Kim, Y., Li, L., Huang, T.H.M., Nephew, K.P., and Kim, S. (2008). Enriched transcription factor binding sites in hypermethylated gene promoters in drug resistant cancer cells. Bioinformatics *24*, 1745–1748. https://doi.org/10.1093/bioinformatics/btn256.

39. Andersson, R., and Sandelin, A. (2020). Determinants of enhancer and promoter activities of regulatory elements. Nat. Rev. Genet. *21*, 71–87. https://doi.org/10.1038/s41576-019-0173-8.

40. Topfer, S.K., Feng, R., Huang, P., Ly, L.C., Martyn, G.E., Blobel, G.A., Weiss, M.J., Quinlan, K.G.R., and Crossley, M. (2022). Disrupting the adult globin promoter alleviates promoter competition and reactivates fetal globin gene expression. Blood *139*, 2107–2118.

41. Cheng, L., Zhang, S., Wang, M., and Lopez-Beltran, A. (2023). Biological and clinical perspectives of TERT promoter mutation detection on bladder cancer diagnosis and management. Hum. Pathol. *133*, 56–75. https://doi.org/10.1016/j.humpath.2022.06.005.

42. Yan, R., Cigliola, V., Oonk, K.A., Petrover, Z., DeLuca, S., Wolfson, D.W., Vekstein, A., Mendiola, M.A., Devlin, G., Bishawi, M., et al. (2023). An enhancer-based gene-therapy strategy for spatiotemporal control of cargoes during tissue repair. Cell Stem Cell *30*, 96–111.e6. https://doi.org/10.1016/j.stem.2022.11.012.

43. Wakita, S., Sakaguchi, M., Oh, I., Kako, S., Toya, T., Najima, Y., Doki, N., Kanda, J., Kuroda, J., Mori, S., et al. (2022). Prognostic impact of *CEBPA* bZIP domain mutation in acute myeloid leukemia. Blood Adv. *6*, 238–247. https://doi.org/10.1182/bloodadvances.2021004292.

44. Durbin, A.D., Wang, T., Wimalasena, V.K., Zimmerman, M.W., Li, D., Dharia, N.V., Mariani, L., Shendy, N.A.M., Nance, S., Patel, A.G., et al. (2022). EP300 Selectively Controls the Enhancer Landscape of *MYCN*-Amplified Neuroblastoma. Cancer Discov. *12*, 730–751. https://doi.org/10.1158/2159-8290.Cd-21-0385.

45. Wang, H., Fan, Z., Shliaha, P.V., Miele, M., Hendrickson, R.C., Jiang, X., and Helin, K. (2023). H3K4me3 regulates RNA polymerase II promoter-proximal pause-release. Nature *615*, 339–348. https://doi.org/10.1038/s41586-023-05780-8.

46. Gao, T., and Qian, J. (2020). EnhancerAtlas 2.0: an updated resource with enhancer annotation in 586 tissue/cell types across nine species. Nucleic Acids Res. *48*, D58–D64. https://doi.org/10.1093/nar/gkz980.

47. Zhang, T., Zhang, Z., Dong, Q., Xiong, J., and Zhu, B. (2020). Histone H3K27 acetylation is dispensable for enhancer activity in mouse embryonic stem cells. Genome Biol. *21*, 45. https://doi.org/10.1186/s13059-020-01957-w.

48. Aran, D., Looney, A.P., Liu, L., Wu, E., Fong, V., Hsu, A., Chak, S., Naikawadi, R.P., Wolters, P.J., Abate, A.R., et al. (2019). Reference-based analysis of lung single-cell sequencing reveals a transitional profibrotic macrophage. Nat. Immunol. *20*, 163–172. https://doi.org/10.1038/s41590-018-0276-y.

49. Gutierrez-Cuevas, J., Santos, A., and Armendariz-Borunda, J. (2021). Pathophysiological Molecular Mechanisms of Obesity: A Link between MAFLD and NASH with Cardiovascular Diseases. Int. J. Mol. Sci. *22*, 11629. https://doi.org/10.3390/ijms222111629.

50. Awan, F.M., Naz, A., Obaid, A., Ali, A., Ahmad, J., Anjum, S., and Janjua, H.A. (2015). Identification of Circulating Biomarker Candidates for Hepatocellular Carcinoma (HCC): An Integrated Prioritization Approach. PLoS One *10*, e0138913. https://doi.org/10.1371/journal.pone.0138913.

51. Dietrich, S., Floegel, A., Weikert, C., Prehn, C., Adamski, J., Pischon, T., Boeing, H., and Drogan, D. (2016). Identification of Serum Metabolites Associated With Incident Hypertension in the European Prospective Investigation into Cancer and Nutrition-Potsdam Study. Hypertension *68*, 471–477. https://doi.org/10.1161/hypertensionaha.116.07292.

52. Meierhofer, D., Halbach, M., Şen, N.E., Gispert, S., and Auburger, G. (2016). Ataxin-2 (Atxn2)-Knock-Out Mice Show Branched Chain Amino Acids and Fatty Acids Pathway Alterations. Mol. Cell. Proteomics *15*, 1728–1739. https://doi.org/10.1074/mcp.M115.056770.

53. Cantara, S., Dalmiglio, C., Marzocchi, C., Sagnella, A., Brilli, L., Trimarchi, A., Maino, F., Valerio, L., and Castagna, M.G. (2023). Pilot Study on the Impact of Polymorphisms Linked to Multi-Kinase Inhibitor Metabolism on Lenvatinib Side Effects in Patients with Advanced Thyroid Cancer. Int. J. Mol. Sci. *24*, 5496. https://doi.org/10.3390/ijms24065496.

54. Zhao, X., Zhang, C., Zhu, L., Wu, B., Han, Y., Heung, M., and Zuo, L. (2021). Severe secondary hyperkalemia and arrhythmia from drug interactions between calcium-channel blocker and voriconazole: a case presentation. BMC Nephrol. *22*, 172. https://doi.org/10.1186/s12882-021-02370-6.

55. Taghvaei, S., Saremi, L., and Babaniamansour, S. (2021). Computational Analysis of Gly482Ser Single-Nucleotide Polymorphism in PPARGC1A Gene Associated with CAD, NAFLD, T2DM, Obesity, Hypertension, and Metabolic Diseases. PPAR Res. *2021*, 5544233. https://doi.org/10.1155/2021/5544233.

56. Gomo, C., Coriat, R., Faivre, L., Mir, O., Ropert, S., Billemont, B., Dauphin, A., Tod, M., Goldwasser, F., and Blanchet, B. (2011). Pharmacokinetic interaction involving sorafenib and the calcium-channel blocker felodipine in a patient with hepatocellular carcinoma. Invest. New Drugs *29*, 1511–1514. https://doi.org/10.1007/s10637-010-9514-3.

57. Sun, X., Tang, S., Hou, B., Duan, Z., Liu, Z., Li, Y., He, S., Wang, Q., and Chang, Q. (2021). Overexpression of P-glycoprotein, MRP2, and CYP3A4 impairs intestinal absorption of octreotide in rats with portal hypertension. BMC Gastroenterol. *21*, 2. https://doi.org/10.1186/s12876-020-01532-4.

58. Qiu, X., Mao, Q., Tang, Y., Wang, L., Chawla, R., Pliner, H.A., and Trapnell, C. (2017). Reversed graph embedding resolves complex single-cell trajectories. Nat. Methods *14*, 979–982. https://doi.org/10.1038/nmeth.4402.

59. Bergen, V., Lange, M., Peidli, S., Wolf, F.A., and Theis, F.J. (2020). Generalizing RNA velocity to transient cell states through dynamical modeling. Nat. Biotechnol. *38*, 1408–1414. https://doi.org/10.1038/s41587-020-0591-3.

60. Granja, J.M., Corces, M.R., Pierce, S.E., Bagdatli, S.T., Choudhry, H., Chang, H.Y., and Greenleaf, W.J. (2021). ArchR is a scalable software package for integrative single-cell chromatin accessibility analysis. Nat. Genet. *53*, 403–411. https://doi.org/10.1038/s41588-021-00790-6.

61. Wong, Y.Y., Harbison, J.E., Hope, C.M., Gundsambuu, B., Brown, K.A., Wong, S. W., Brown, C.Y., Couper, J.J., Breen, J., Liu, N., et al. (2023). Parallel recovery of chromatin accessibility and gene expression dynamics from frozen human regulatory T cells. Sci. Rep. *13*, 5506. https://doi.org/10.1038/s41598-023-32256-6.

62. Aygun, N., Liang, D., Crouse, W.L., Keele, G.R., Love, M.I., and Stein, J.L. (2023). Inferring cell-type-specific causal gene regulatory networks during human neurogenesis. Genome Biol. *24*, 130. https://doi.org/10.1186/s13059-023-02959-0.

63. Tu, Z., Bi, Y., Mao, T., Wang, H., Gao, S., and Wang, Y. (2023). Discordance between chromatin accessibility and transcriptional activity during the human primed-to-naive pluripotency transition process. Cell Regen. *12*, 35. https://doi.org/10.1186/s13619-023-00179-2.

64. Iacob, S., Iacob, R., Manea, I., Uta, M., Chiosa, A., Dumbrava, M., Becheanu, G., Stoica, L., Popa, C., Brasoveanu, V., et al. (2022). Host and immunosuppression-related factors influencing fibrosis occurrence post liver transplantation. Front. Pharmacol. *13*, 1042664. https://doi.org/10.3389/fphar.2022.1042664.

65. Berasain, C., Herrero, J.I., García-Trevijano, E.R., Avila, M.A., Esteban, J.I., Mato, J. M., and Prieto, J. (2003). Expression of Wilms' tumor suppressor in the liver with cirrhosis:: Relation to hepatocyte nuclear factor 4 and hepatocellular function. Hepatology *38*, 148–157. https://doi.org/10.1053/jhep.2003.50269.

66. Drzewiecki, K., Choi, J., Brancale, J., Leney-Greene, M.A., Sari, S., Dalgiç, B., Ünlüsoy Aksu, A., Evirgen Şahin, G., Ozen, A., Baris, S., et al. (2021). GIMAP5 maintains liver endothelial cell homeostasis and prevents portal hypertension. J. Exp. Med. *218*, e20201745. https://doi.org/10.1084/jem.20201745.

67. Bravo González-Blas, C., De Winter, S., Hulselmans, G., Hecker, N., Matetovici, I., Christiaens, V., Poovathingal, S., Wouters, J., Aibar, S., and Aerts, S. (2023). SCENIC+: single-cell multiomic inference of enhancers and gene regulatory networks. Nat. Methods *20*, 1355–1367. https://doi.org/10.1038/s41592-023-01938-4.

68. Fishilevich, S., Nudel, R., Rappaport, N., Hadar, R., Plaschkes, I., Iny Stein, T., Rosen, N., Kohn, A., Twik, M., Safran, M., et al. (2017). GeneHancer: genome-wide integration of enhancers and target genes in GeneCards. Database *2017*, bax028. https://doi.org/10.1093/database/bax028.

69. van Kuilenburg, A.B.P., Meinsma, R., Beke, E., Assmann, B., Ribes, A., Lorente, I., Busch, R., Mayatepek, E., Abeling, N.G.G.M., van Cruchten, A., et al. (2004). β--ureidopropionase deficiency:: an inborn error of pyrimidine degradation associated with neurological abnormalities. Hum. Mol. Genet. *13*, 2793–2801. https://doi.org/10.1093/hmg/ddh303.

70. Li, B., Feng, W., Luo, O., Xu, T., Cao, Y., Wu, H., Yu, D., and Ding, Y. (2017). Development and Validation of a Three-gene Prognostic Signature for Patients with Hepatocellular Carcinoma. Sci. Rep. *7*, 5517. https://doi.org/10.1038/s41598-017-04811-5.

71. Chen, Y., Ying, Y., Ma, W., Ma, H., Shi, L., Gao, X., Jia, M., Li, M., Song, X., Kong, W., et al. (2024). Targeting the Epigenetic Reader ENL Inhibits Super-Enhancer-Driven Oncogenic Transcription and Synergizes with BET Inhibition to Suppress Tumor Progression. Cancer Res. *84*, 1237–1251. https://doi.org/10.1158/0008-5472.Can-23-1836.

72. Gosai, S.J., Castro, R.I., Fuentes, N., Butts, J.C., Mouri, K., Alasoadura, M., Kales, S., Nguyen, T.T.L., Noche, R.R., Rao, A.S., et al. (2024). Machine-guided design of cell-type-targeting *cis*-regulatory elements. Nature *634*, 1211–1220. https://doi.org/10.1038/s41586-024-08070-z.

73. Caragine, C.M., Le, V.T., Mustafa, M., Diaz, B.J., Morris, J.A., Müller, S., Mendez-Mancilla, A., Geller, E., Liscovitch-Brauer, N., and Sanjana, N.E. (2025). Comprehensive dissection of *cis*-regulatory elements in a 2.8 Mb topologically associated domain in six human cancers. Nat. Commun. *16*, 1611. https://doi.org/10.1038/s41467-025-56568-5.

74. Reilly, S.K., Gosai, S.J., Gutierrez, A., Mackay-Smith, A., Ulirsch, J.C., Kanai, M., Mouri, K., Berenzy, D., Kales, S., Butler, G.M., et al. (2021). Direct characterization of *cis*-regulatory elements and functional dissection of complex genetic associations using HCR-FlowFISH. Nat. Genet. *53*, 1166–1176. https://doi.org/10.1038/s41588-021-00900-4.

75. Hao, Y., Stuart, T., Kowalski, M.H., Choudhary, S., Hoffman, P., Hartman, A., Srivastava, A., Molla, G., Madad, S., Fernandez-Granda, C., and Satija, R. (2024). Dictionary learning for integrative, multimodal and scalable single-cell analysis. Nat. Biotechnol. *42*, 293–304. https://doi.org/10.1038/s41587-023-01767-y.

76. Quinlan, A.R., and Hall, I.M. (2010). BEDTools: a flexible suite of utilities for comparing genomic features. Bioinformatics *26*, 841–842. https://doi.org/10.1093/bioinformatics/btq033.

77. Ramírez, F., Dündar, F., Diehl, S., Grüning, B.A., and Manke, T. (2014). deepTools: a flexible platform for exploring deep-sequencing data. Nucleic Acids Res. *42*, W187–W191. https://doi.org/10.1093/nar/gku365.

78. ENCODE Project Consortium (2012). An integrated encyclopedia of DNA elements in the human genome. Nature *489*, 57–74. https://doi.org/10.1038/nature11247.

79. Luo, Y., Hitz, B.C., Gabdank, I., Hilton, J.A., Kagda, M.S., Lam, B., Myers, Z., Sud, P., Jou, J., Lin, K., et al. (2020). New developments on the Encyclopedia of DNA Elements (ENCODE) data portal. Nucleic Acids Res. *48*, D882–D889. https://doi.org/10.1093/nar/gkz1062.

OMTN, Volume *36*

Supplemental information

Single-cell multi-omics data reveal heterogeneity in liver tissue microenvironment induced by hypertension

Hongfei Li, Lingyu Cui, Murong Zhou, Quan Zou, Yuming Zhao, Hao Lin, Yingjian Liang, Alfred Wei Chieh Kow, and Guohua Wang

Supplemental Figures

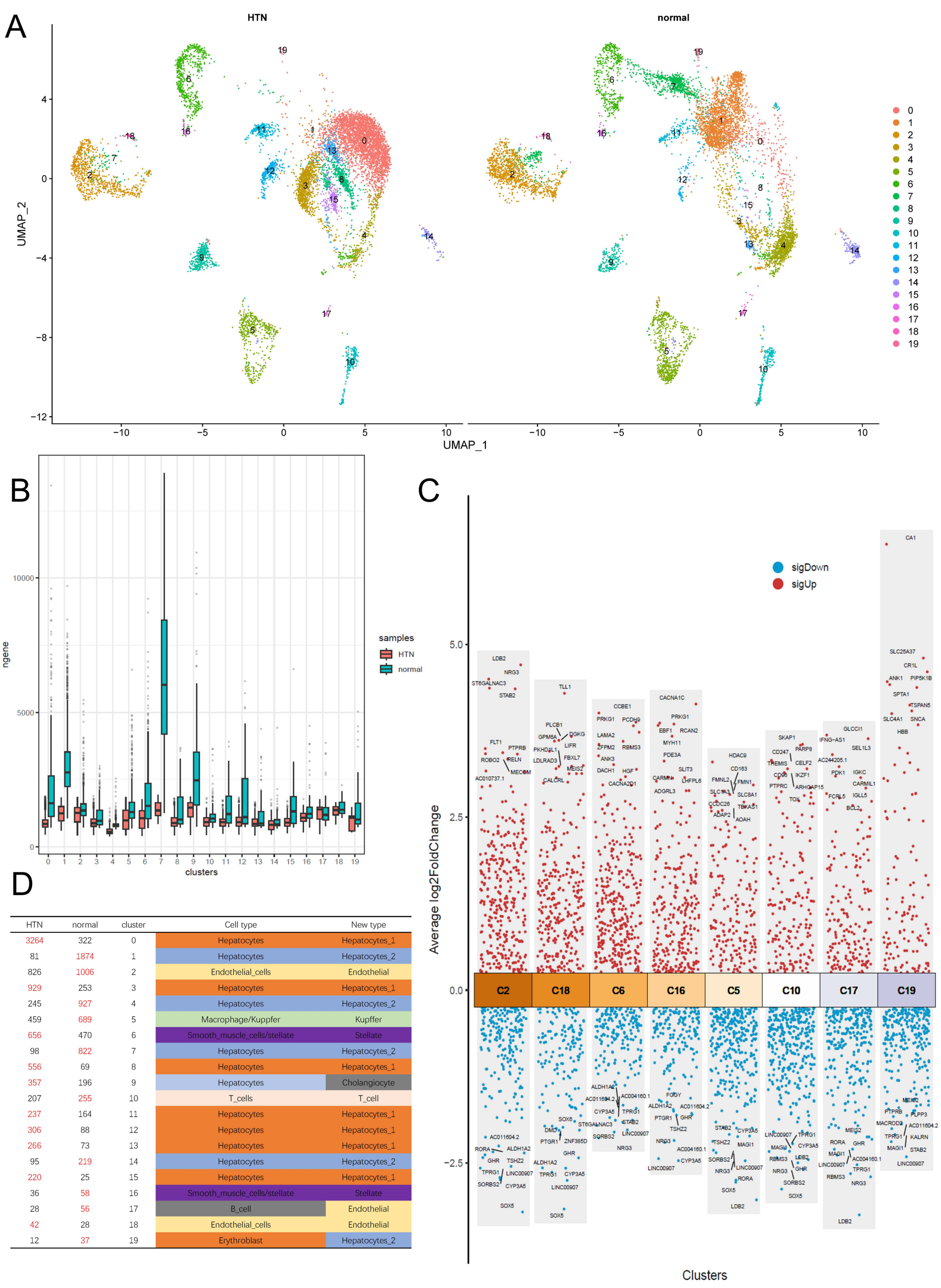

HTN	normal	cluster	Cell type	New type
3264	322	0	Hepatocytes	Hepatocytes_1
81	1874	1	Hepatocytes	Hepatocytes_2
826	1006	2	Endothelial_cells	Endothelial
929	253	3	Hepatocytes	Hepatocytes_1
245	927	4	Hepatocytes	Hepatocytes_2
459	689	5	Macrophage/Kuppfer	Kupffer
656	470	6	Smooth_muscle_cells/stellate	Stellate
98	822	7	Hepatocytes	Hepatocytes_2
556	69	8	Hepatocytes	Hepatocytes_1
357	196	9	Hepatocytes	Cholangiocyte
207	255	10	T_cells	T_cell
237	164	11	Hepatocytes	Hepatocytes_1
306	88	12	Hepatocytes	Hepatocytes_1
266	73	13	Hepatocytes	Hepatocytes_1
95	219	14	Hepatocytes	Hepatocytes_2
220	25	15	Hepatocytes	Hepatocytes_1
36	58	16	Smooth_muscle_cells/stellate	Stellate
28	56	17	B_cell	Endothelial
42	28	18	Endothelial_cells	Endothelial
12	37	19	Erythroblast	Hepatocytes_2

Figure S1. Heterogeneity of scRNA clusters

(A) UAMP of cell clustering for HTN and normal cells.

(B) Variation in the number of genes.

(C) Differential genes in hepatocyte subsets were excluded.

(D) Cell annotation based on singeR and manual.

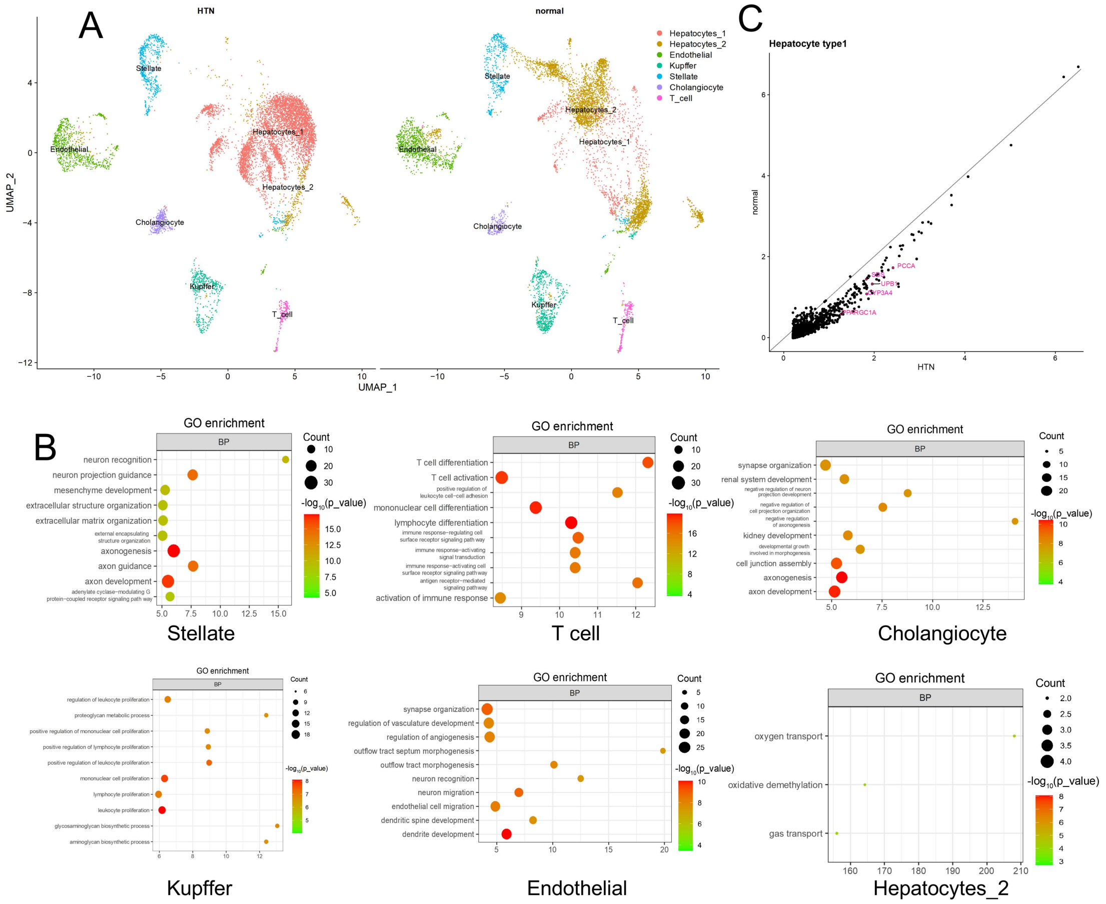

Figure S2. Functional enrichment of cell subsets

(A) UAMP of cell subsets for HTN and normal cells based on manual annotation.

(B) Enrichment of GO function in cell subsets.

(C) Differential expression of marker genes in hepatocytes_1.

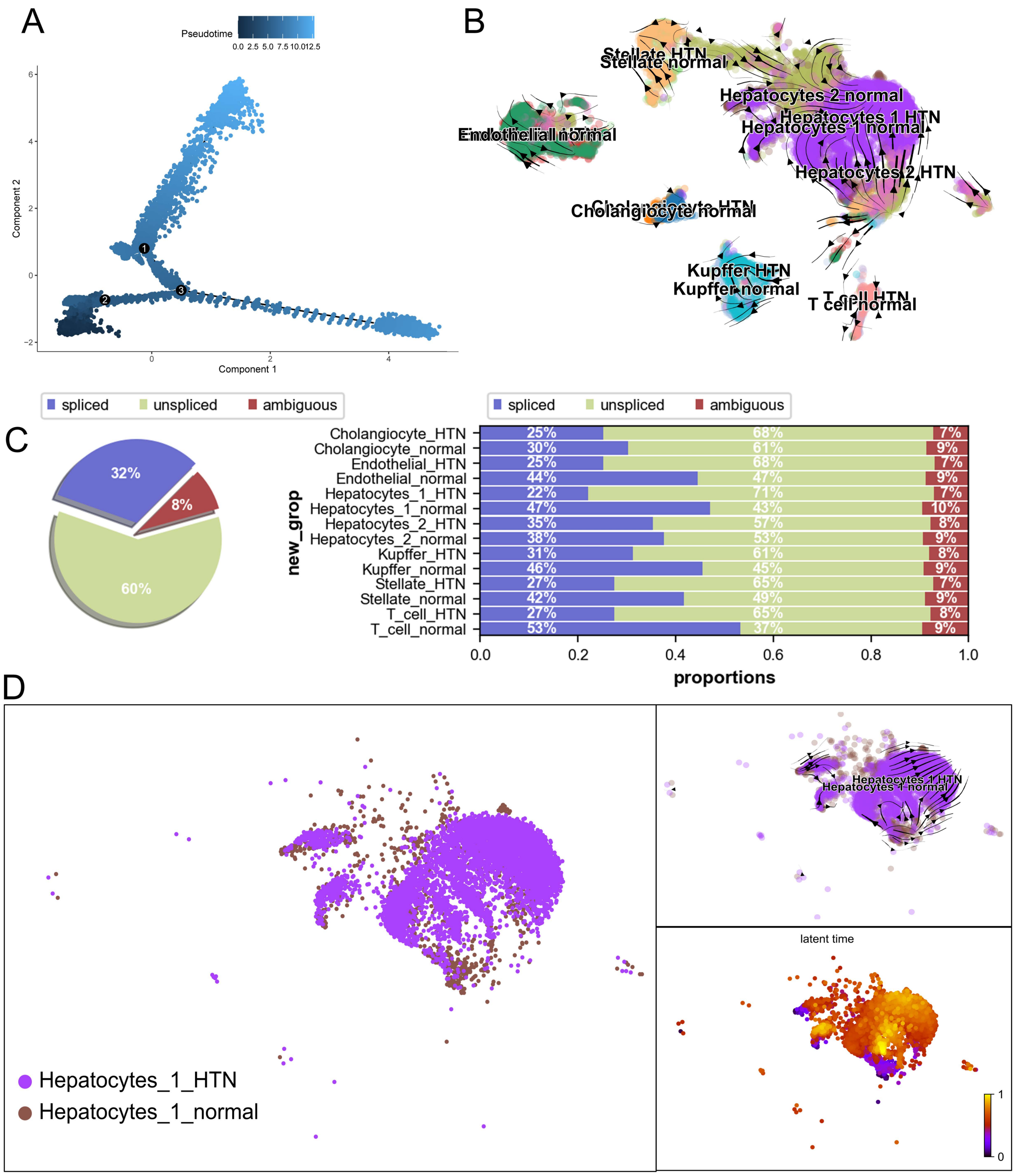

Figure S3. Validation of trajectory inference was performed using scVelo

(A) The pseudotime for trajectory inference in Monocle2.

(B). The RNA velocity trends of all cell types.

(C). The splicing states of all cell types.

(D). RNA Velocity Reveals the Directionality of Hepatocyte Differentiation Trajectory.

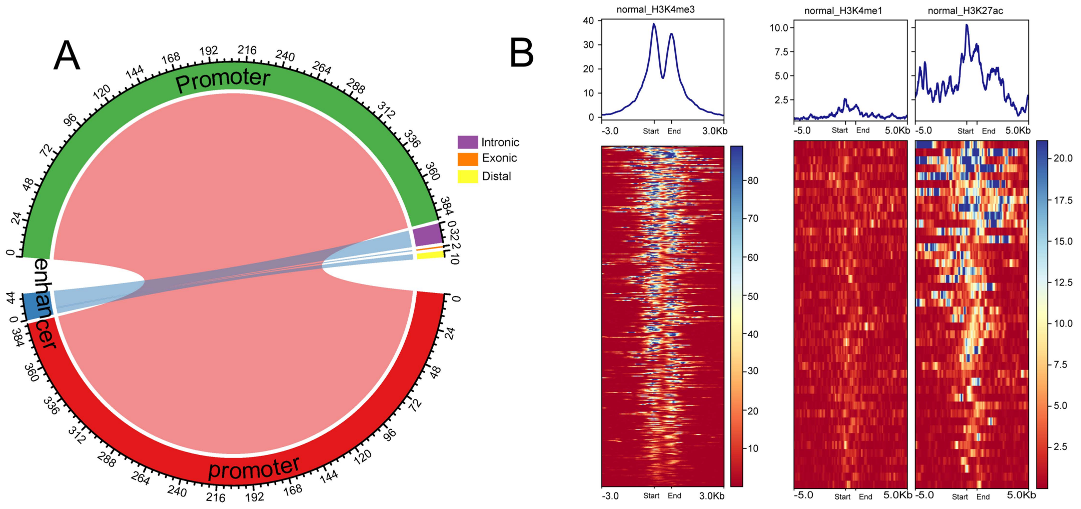

Figure S4. Predicting promoters of enhancers and promoters for normal Hepatocytes_1

(A) Annotation of promoter and enhancer for normal Hepatocytes_1.

(B). The distribution of histone signals in the promoter and enhancer regions of normal Hepatocytes_1.

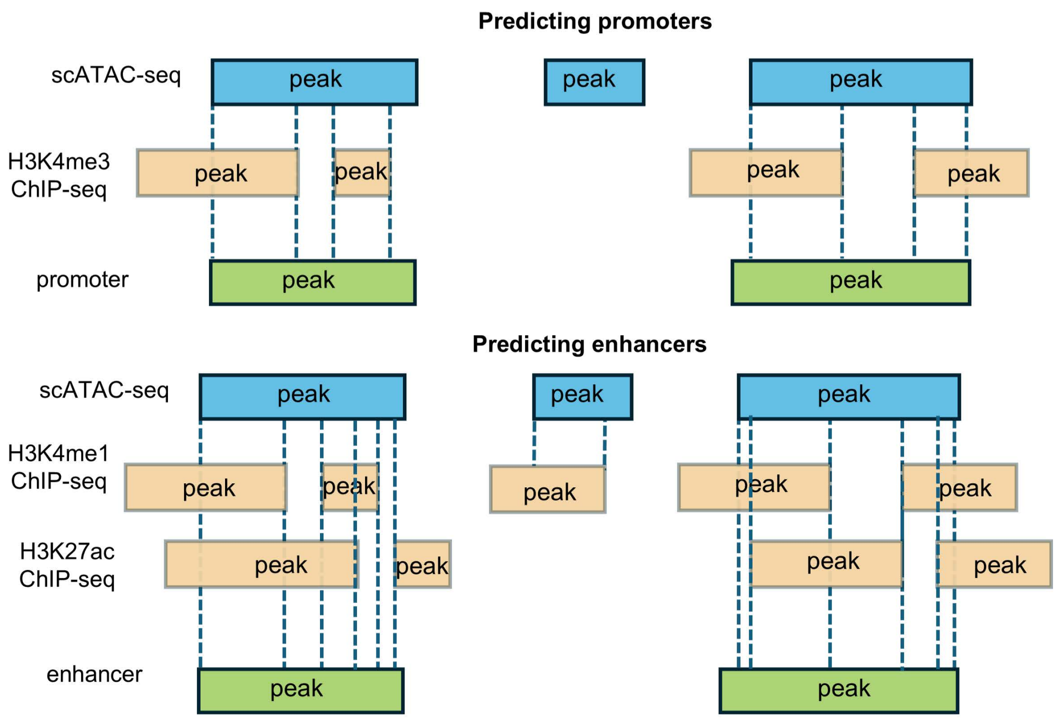

Figure S5. Predicting promoters and enhancers by overlapping peaks from scATAC-seq and histone ChIP-seq data.

Supplemental Tables

Table S1. The result of clusters and their corresponding cell numbers from the initial clustering of scRNA-seq.

HTN	normal	cluster	Percentage
3264	322	0	0.216663646
81	1874	1	0.118119751
826	1006	2	0.110688176
929	253	3	0.071415624
245	927	4	0.070811431
459	689	5	0.069361368
656	470	6	0.068032143
98	822	7	0.055585765
556	69	8	0.037762069
357	196	9	0.033411878
207	255	10	0.027913721
237	164	11	0.024228143
306	88	12	0.023805208
266	73	13	0.020482146
95	219	14	0.018971663
220	25	15	0.014802731
36	58	16	0.005679415
28	56	17	0.005075222
42	28	18	0.004229352
12	37	19	0.002960546

Table S2. Differential genes from the initial clustering of scRNA-seq (see Excel file).

Table S3. Average expression values of 2,000 genes in the finally annotated cell types (see Excel file).

Table S4. The number of clusters and corresponding cell counts from the annotated cell types of scRNA-seq.

HTN	normal	cluster
5778	994	Hepatocytes_1
531	3879	Hepatocytes_2
896	1090	Endothelial
459	689	Kupffer
692	528	Stellate
357	196	Cholangiocyte
207	255	T_cell

Table S5. Differential genes from the finally annotated cell types in scRNA-seq (see Excel file).

Table S6. The enriched GO pathways based on upregulated genes (avg_log2FC > 0 and p_val < 0.05) of Hepatocytes_1 base on Table S5.

ONTOLOGY	ID	Description	GeneRatio	BgRatio	pvalue	p.adjust	qvalue	geneID	Count
BP	GO:0006631	fatty acid metabolic process	10/67	390/18723	0.00000118	0.00198	0.001573337	ECHDC2/PTGR1/PCCA/CYP3A4/ PLIN5/PNPLA3/CYP2C19/PPARGC1A/PCK1/MLXIPL	10
BP	GO:0044282	small molecule catabolic process	9/67	376/18723	0.00000739	0.00413	0.00328205	ECHDC2/PCCA/KMO/CYP3A4 /UPB1/PLIN5/GOT1/SDS/PCK1	9
BP	GO:0042445	hormone metabolic process	7/67	218/18723	0.0000127	0.004625	0.003675628	ALDH1A2/GHR/CYP3A5/PCSK6/ SLC16A10/CYP3A4/PPARGC1A	7
BP	GO:0046394	carboxylic acid biosynthetic process	8/67	314/18723	0.0000158	0.004625	0.003675628	ALDH1A2/KMO/NR1H4/CYP3A4/ UPB1/GOT1/SDS/MLXIPL	8
BP	GO:0016053	organic acid biosynthetic process	8/67	316/18723	0.0000165	0.004625	0.003675628	ALDH1A2/KMO/NR1H4/CYP3A4/ UPB1/GOT1/SDS/MLXIPL	8
BP	GO:0046395	carboxylic acid catabolic process	7/67	236/18723	0.0000211	0.004695	0.003730857	ECHDC2/PCCA/KMO/ PLIN5/GOT1/SDS/PCK1	7
BP	GO:0016054	organic acid catabolic process	7/67	240/18723	0.0000236	0.004695	0.003730857	ECHDC2/PCCA/KMO/ PLIN5/GOT1/SDS/PCK1	7
BP	GO:0006721	terpenoid metabolic process	5/67	97/18723	0.0000252	0.004695	0.003730857	ALDH1A2/CYP3A5/ EGFR/CYP3A4/CYP2C19	5
BP	GO:0006090	pyruvate metabolic process	5/67	106/18723	0.0000387	0.005693	0.004523819	SLC4A4/SDS/ PPARGC1A/PCK1/MLXIPL	5
BP	GO:0071466	cellular response to xenobiotic stimulus	6/67	177/18723	0.0000406	0.005693	0.004523819	RORA/CYP3A5/EGFR/ UGT1A10/CYP3A4/CYP2C19	6
BP	GO:0006805	xenobiotic metabolic process	5/67	111/18723	0.0000482	0.00622	0.004942357	RORA/CYP3A5/UGT1A10/ CYP3A4/CYP2C19	5
BP	GO:0006720	isoprenoid metabolic process	5/67	116/18723	0.0000595	0.007129	0.00566472	ALDH1A2/CYP3A5/EGFR/ CYP3A4/CYP2C19	5
BP	GO:0071375	cellular response to peptide hormone stimulus	7/67	290/18723	0.0000782	0.00874	0.006945063	GHR/BRIP1/ERRFI1/GOT1/ PNPLA3/TSHR/PCK1	7

Table S7. Differential genes between HTN and normal states of Hepatocytes_1 (see Excel file).

Table S8. The numbers of the four types of peaks in Hepatocytes_1 under HTN and normal conditions.

Peak type	normal	HTN
Distal	2547	1586
Exonic	500	717
Intronic	2993	3147
Promoter	547	1102
Total	6587	6552

Table S9. Predicted promoters of Hepatocytes_1 in the HTN state (see Excel file).

Table S10. Predicted enhancers of Hepatocytes_1 in the HTN state (see Excel file).

Table S11. Predicted promoters of Hepatocytes_1 in the normal state (see Excel file).

Table S12. Predicted enhancers of Hepatocytes_1 in the normal state.

Chromosome	Start	End	Peak ID	Gene	ArchR-annotated peak types
chr1	39927586	39928086	4215	MYCL	Distal
chr1	40383720	40384220	4274	SMAP2	Intronic
chr1	201512891	201513391	12449	CSRP1	Distal
chr2	73032654	73033154	20384	SFXN5	Intronic
chr2	177239905	177240405	25213	MIR3128	Intronic
chr2	224379425	224379925	27479	FAM124B	Exonic
chr3	46868180	46868680	31630	PTH1R	Intronic
chr3	113686109	113686609	34639	USF3	Intronic
chr4	6918130	6918630	40200	TBC1D14	Intronic
chr4	184345571	184346071	46757	LINC0236	Intronic
chr5	67547321	67547821	49779	LOC10192	Distal
chr5	150515886	150516386	53991	NDST1-AS	Intronic
chr5	150589369	150589869	54011	SYNPO	Distal
chr5	151085368	151085868	54084	TNIP1	Intronic
chr5	176534709	176535209	55389	RNF44	Intronic
chr6	53301237	53301737	60078	MIR5685	Intronic
chr6	169905609	169906109	65247	LINC0024	Distal
chr9	38389178	38389678	83112	ALDH1B1	Distal
chr9	104911079	104911579	85349	ABCA1	Intronic
chr9	129570304	129570804	87510	NTMT1	Distal
chr10	102280120	102280620	95426	GBF1	Intronic
chr11	86940924	86941424	103312	FZD4	Intronic
chr12	29241644	29242144	107514	LOC10050	Intronic
chr12	50304106	50304606	108343	LIMA1	Distal
chr12	111777634	111778134	111708	MIR6761	Intronic
chr13	97257670	97258170	115927	MBNL2	Intronic
chr13	110507025	110507525	116556	COL4A2-A	Intronic
chr15	63211400	63211900	124029	RAB8B	Intronic
chr15	78880941	78881441	125381	CTSH	Intronic
chr15	90035450	90035950	126062	MIR3174	Intronic
chr16	79970435	79970935	131723	MAFTRR	Distal
chr16	81552895	81553395	131848	MIR7854	Intronic
chr17	29153763	29154263	135200	MYO18A	Intronic
chr17	40108171	40108671	136063	NR1D1	Distal
chr17	64085049	64085549	138108	ERN1	Intronic
chr17	64156000	64156500	138128	SNORA50	Intronic
chr18	44725573	44726073	142172	SETBP1	Intronic
chr19	7258252	7258752	145241	INSR	Intronic
chr19	15325110	15325610	146300	BRD4	Intronic
chr19	18496409	18496909	146862	ELL	Intronic
chr19	38837709	38838209	147918	ECH1	Intronic
chr19	46975156	46975656	148960	NPAS1	Intronic
chr22	37503824	37504324	158423	CARD10	Exonic
chr22	46058088	46058588	159505	PRR34	Intronic

Table S13. Regulatory network of promoters of upregulated genes in HTN-Hepatocytes_1.

Promoter (peak ID)	Gene	TF
58694	ANKS1A	EBF1
58694	ANKS1A	WT1
58694	ANKS1A	ETS1
58694	ANKS1A	EHF
58694	ANKS1A	FLI1
58694	ANKS1A	ELF3
58694	ANKS1A	PAX2
70606	CYP3A4	FOXP1
70606	CYP3A4	GATA4
70606	CYP3A4	PBX1
70606	CYP3A4	RORA
70606	CYP3A4	RORB
85666	PALM2-AKAP2	WT1
131100	PDXDC2P-NPIPB14P	EBF1
131100	PDXDC2P-NPIPB14P	WT1
131100	PDXDC2P-NPIPB14P	ETS1
131100	PDXDC2P-NPIPB14P	EHF
131100	PDXDC2P-NPIPB14P	FLI1
131100	PDXDC2P-NPIPB14P	ELF3
131210	TAT-AS1	EBF1
131210	TAT-AS1	MEF2C
131210	TAT-AS1	RARB
131210	TAT-AS1	TCF7L1
136475	G6PC	HNF1B
136475	G6PC	RORA
136475	G6PC	TCF7L1

Table S14. Regulatory network of enhancers of upregulated genes in HTN-Hepatocytes_1.

Region	Gene	TF	Peak ID	data_attributes
chr13:94157698-94158198	GPC6	CEBPD	115803	genehancer_id=GH13F094157
chr22:37178198-37178698	IL2RB	E2F8	158352	genehancer_id=GH22F037175
chr22:24510675-24511175	UPB1	NR1H4	157194	genehancer_id=GH22F024510
chr22:24510675-24511175	UPB1	THRB	157194	genehancer_id=GH22F024510
chr5:150142139-150142639	CSF1R	NR1H4	53922	genehancer_id=GH05F150133
chr5:150142139-150142639	PDGFRB	THRB	53922	genehancer_id=GH05F150133
chr3:43317938-43318438	SNRK	NR2F1	31315	genehancer_id=GH03F043316
chr3:43317938-43318438	SNRK	NR2F2	31315	genehancer_id=GH03F043316
chr3:43317938-43318438	SNRK	THRB	31315	genehancer_id=GH03F043316
chr9:35834520-35835020	TPM2	NR2F1	82894	genehancer_id=GH09F035831
chr5:157288526-157289026	ITK	NR2F2	54386	genehancer_id=GH05F157287
chr5:157288526-157289026	ITK	PPARG	54386	genehancer_id=GH05F157287
chr10:112261401-112261901	GPAM	PPARA	96006	genehancer_id=GH10F112259
chr8:124630983-124631483	MTSS1	ZNF385D	79770	genehancer_id=GH08F124625
chr10:88840070-88840570	LIPN	ELK3	94265	genehancer_id=GH10F088840
chr6:39280222-39280722	KCNK17	PRRX1	59150	genehancer_id=GH06F039279
chr12:113246398-113246898	TPCN1	NR2E3	111827	genehancer_id=GH12F113235
chr2:102120584-102121084	IL1R1	ETS1	21784	genehancer_id=GH02F102120
